# Solutions of Critical Raw Materials Issues Regarding Iron-Based Alloys

**DOI:** 10.3390/ma14040899

**Published:** 2021-02-13

**Authors:** Pavel Novák, Tiziano Bellezze, Marcello Cabibbo, Ernst Gamsjäger, Manfred Wiessner, Dragan Rajnovic, Lucyna Jaworska, Pavel Hanus, Andrei Shishkin, Gaurav Goel, Saurav Goel

**Affiliations:** 1 Department of Metals and Corrosion Engineering, University of Chemistry and Technology, Prague, Technická 5, 166 28 Prague, Czech Republic; 2 Department of Materials, Environmental Sciences and Urban Planning, Polytechnic University of Marche, Via Brecce Bianche, 60131 Ancona, Italy; t.bellezze@univpm.it; 3 Dipartimento di Ingegneria Industriale e Scienze Matematiche (DIISM), Università Politecnica delle Marche, 60131 Ancona, Italy; m.cabibbo@staff.univpm.it; 4 Institute of Mechanics, Montanuniversität Leoben, Franz-Josef-Str. 18, 8700 Leoben, Austria; e.gamsjaeger@unileoben.ac.at; 5 Anton Paar GmbH, Anton-Paar-Str. 20, 8054 Graz, Austria; manfred.wiessner@anton-paar.com; 6 Department of Production Engineering, Faculty of Technical Science, University of Novi Sad, Novi Sad, Trg Dositeja Obradovica 6, 21000 Novi Sad, Serbia; draganr@uns.ac.rs; 7 Faculty of Non-Ferrous Metals, AGH University of Science and Technology, 30-059 Krakow, Poland; ljaw@agh.edu.pl; 8 Department of Material Science, Faculty of Mechanical Engineering, Technical University of Liberec, 461 17 Liberec, Czech Republic; pavel.hanus@tul.cz; 9 Rudolfs Cimdins Riga Biomaterials Innovations and Development Centre of RTU, Faculty of Materials Science and Applied Chemistry, Riga Technical University, Pulka 3, LV-1007 Riga, Latvia; andrejs.siskins@rtu.lv; 10 School of Engineering, London South Bank University, 103 Borough Road, London SE1 0AA, UK; Goelg@Lsbu.ac.uk (G.G.); saurav.goel@cranfield.ac.uk (S.G.); 11 School of Aerospace, Transport and Manufacturing, Cranfield University, Cranfield MK43 0AL, UK

**Keywords:** critical raw materials, substitution, iron, alloy

## Abstract

The Critical Raw Materials (CRMs) list has been defined based on economic importance and supply risk by the European Commission. This review paper describes two issues regarding critical raw materials: the possibilities of their substitution in iron-based alloys and the use of iron-based alloys instead of other materials in order to save CRMs. This review covers strategies for saving chromium in stainless steel, substitution or lowering the amounts of carbide-forming elements (especially tungsten and vanadium) in tool steel and alternative iron-based CRM-free and low-CRM materials: austempered ductile cast iron, high-temperature alloys based on intermetallics of iron and sintered diamond tools with an iron-containing low-cobalt binder.

## 1. Introduction

The burgeoning population and increasing industrialization are leading to an increase in pressure on resources. Other factors such as digitalization, the transition to climate neutrality with metals, minerals and biotic materials used in low emission technologies and products and increasing demand from developing countries are also contributing significantly in addition to this pressure. In order to overcome the issue of demand for virgin materials, robust and fundamental change is required in the manufacturing process. Organisation for Economic Co-operation and Development (OECD) forecasts that global materials demand will more than double from 79 billion tons today to 167 billion tons in 2060 [1]. Assuming no change in the current processes would lead to fierce competition among the nations. One such resource is a group of critical raw materials (e.g., arsenic, cadmium, strontium, zirconium), and the continued dependence on them will soon replace today’s dependence on oil. The new industrial strategy of the European Union focuses on this need of integrating secondary raw materials and has embarked upon the journey of the green deal. This ambitious plan to integrate both primary and secondary raw materials, in particular critical raw materials, for key technologies and strategic sectors as renewable energy, e-mobility, digital, space and defense are one of the pre-requisites for achieving climate neutrality [1].

Critical Raw Materials (CRM) are those, which display a particularly high risk of a supply shortage in the next 10 years and which are particularly important for the value chain. Due to the uncertain geopolitical environment, and during the pandemic scenario, supply chains are disrupted considerably. This leads to adverse conditions for industrial productivity of the member nations of the European Union. The supply risk is linked to the concentration of production in a handful of countries, and the risk is in many cases compounded by the low substitutability and low recycling rates of CRMs [2]. The perpetual supply of these critical raw materials is also important for climate policy objectives and for technological innovation. For example, rare earth metals are essential for high-performance permanent magnets in wind turbines or electric vehicles, catalytic converters for cars, printed circuit boards, optical fibers and high-temperature superconductors. Thus, it becomes imperative to either find alternatives to these raw materials or to bring a fundamental shift in the current manufacturing processes.

Concerned about the uninterrupted supply of these raw materials, the European Commission launched the Raw Materials Initiative (RMI) in 2008 [3]. The policy stresses the need to diversify and secure non-energy raw materials for EU industrial value chains. Diversification of supply concerns reducing dependencies in all dimensions by the sourcing of primary raw materials from the EU and third countries, increasing secondary raw materials supply through resource efficiency and circularity and finding alternatives to scarce raw materials.

The methodology presented here is adopted from the European Union report on critical raw materials [4]. The first assessment conducted in 2011 identified 14 critical raw materials (CRMs) out of the 41 non-energy, non-agricultural candidate raw materials. The second exercise in 2014 leads to an increase in these numbers, 20 raw materials out of 54 candidates. The third attempt in 2017 identified more of these critical raw materials, as 27 CRMs were identified among 78 candidates [5]. The latest assessment conducted in 2020 covers a larger number of materials: 83 individual materials or 66 candidate raw materials comprising 63 individual and 3 grouped materials (ten individual heavy rare earth elements (REEs), five-light REEs and five platinum-group metals (PGMs)). Five new materials (arsenic, cadmium, strontium, zirconium and hydrogen) have been assessed [5].

Of the 83 individual (66 candidates) raw materials assessed, the following 30 were identified as critical in this assessment (Table 1)

**Figure 1:**
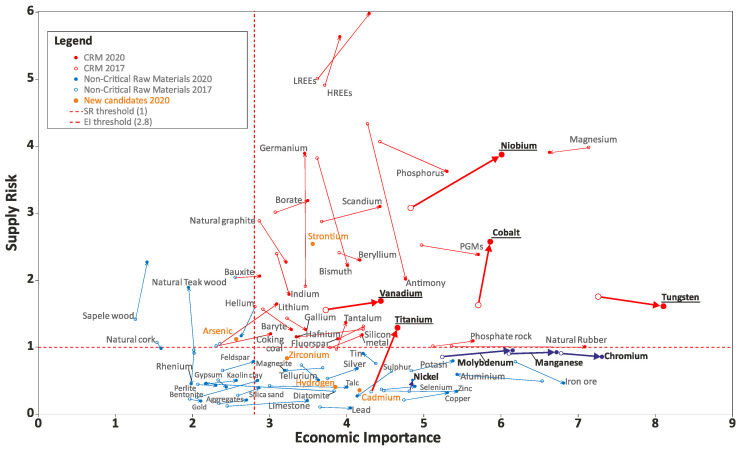
Assessment of the criticality dynamics in 2017–2020 (individual materials and groups), adopted from [5].

The overall results of the 2020 criticality assessment are presented in Figure 1. Critical raw materials (CRMs) are highlighted by red dots and are located within the criticality zone (Supply Risk (SR) ≥ 1 and Economic importance (EI) ≥ 2.8) of the graph. Blue dots represent non-critical raw materials.

The key changes in the 2020 CRMs list compared to the 2014 CRMs list are the 2020 assessment-confirmed 19 CRMs from the 2014 list, whereas 8 (Baryte, Bauxite, Hf, Natural Rubber, Sc, Ta, Ti and V) of the non-critical materials in 2014 shifted to being critical in 2020 [5]. The 2020 CRMs list includes 26 of the CRMs identified in 2017. Only helium, which was listed in 2017, shifted out of the list. Compared to the 2017 CRM list, four additional raw materials (bauxite, Li, Ti and Sr) are identified as critical and enter the 2020 CRMs list [5].

In the CRM list, there are several elements, which are important for the production of the iron-based alloys. Tungsten and vanadium are used as carbide-forming elements in tool steel. Vanadium is usually bound in MC carbides, having a cubic structure. These carbides, due to high thermal stability, arise already in the first stage of crystallization, forming as primary carbides. The presence of these carbides as precipitates was also proved during heat treatment [6]. Tungsten is usually contained in M_6_C primary carbides having a cubic structure, while chromium which was considered as a CRM recently, could be present in “blocky” M_7_C_3_ carbides (hexagonal structure) and M_23_C_6_ (cubic), which could arise during heat treatment. Cobalt is also added to high-speed steel in order to prevent thermal degradation during the service [7].

Among the elements contained in the various grades of stainless steels (SSs), chromium plays the fundamental role in maintaining their corrosion resistance properties in several application fields and in corrosive exposures. At the same time, this element can be considered a critical raw material, although the supply risks (SRs) have decreased in the last six years within the EU from a commercial and industrial standpoint. 

In general, various alloys contain Cr for increasing their corrosion resistance, both in dry and in wet exposure conditions, but SSs are the most important ones within this materials family. They used 74% of the Cr among the different market goods [8], therefore it is considered a pillar of the EU economy. The EU produces 21.1% of worldwide steel output [9], second only to China (45.5%), but it does not have easy access to the raw materials (i.e., Ni, Cr, Mo, etc.) or sufficient resources of Cr required for producing SSs.

Because of this reason, Cr has gained attention during the last decade, becoming a topic of supply and economic analyses, as shown in the EU reports on CRMs from 2014 to 2020 [5,9,10]. It has been found inside the criticality area only in the 2014 report [9] on the SR vs Economic Importance (EI) plot. However, it must be specified that Cr is in the border of this area in the 2014 report and a little bit out of the border in 2017 and 2020 reports, especially in terms of SRs. Furthermore, the result of these analyses is considered “a false impression created by the change in methodology” [11] passing from the old report to the new ones. Therefore, Cr remains nowadays of high strategic value for the EU processing industry and it can still be considered a CRM.

Cr is obtained from chromite ore, refined to become ferrochromium, in particular in the manufacturing of SSs. Concerning the ores, the highest amounts are present in South Africa (46%), Zimbabwe, Kazakhstan and India. Ferrochromium is obtained mainly in China (37%), which dominates the market for this material in recent years for domestic use and exports [8]. Looking at the EU domestic sources, chromite is mainly present in Finland (Kemi mine), while the ferrochromium production is again mainly concentrated in this country, but also in Sweden and Germany.

Taking into account that these EU Cr sources are not sufficient, the import of the above-mentioned materials is of crucial importance, given the EU SSs demand. The main non-EU suppliers, as expected based on the above data, are South Africa and China, but it must be considered that the former is a “critical country” in terms of governance, rule of law, etc. and the latter increased the domestic use over time and applies a 20% export tax. In general, the import of elements necessary for SSs manufacturing coming from non-EU markets is an issue related to uncertain political stability and an exporting policy of these countries. Consequently, the strong dependence of the EU on these market issues can be easily understood, and the SRs of Cr cannot be neglected. This has prompted the EU to find strategies and solutions to save the demand/use of Cr in the production of SSs or other stainless Fe-based alloys but preserve the corrosion resistance, which represents a huge challenge [12].

This review aims to show the ways for:

- The substitution and minimizing of the use of CRMs and chromium, which could be also considered strategic, in iron-based alloys (steel and cast iron);
- The use of iron-based materials to substitute other CRM-containing materials.

## 2. Substitution and Saving of Chromium in Stainless Steel

A possible solution to reduce the consumption of Cr in the obtainment of SSs is material recycling by the use of scraps, which can reach 60% of the industrial production [8], even if it is complicated to sort them from those coming from more common steels, with the consequence of producing only low-performance steels. Considering also the availability of domestic SS scraps and the low recycling rate of Cr [9], it seems to be a limiting strategy to save Cr. On the other hand, considering the increasing demand for SSs, some authors [13] report that the use of scraps is unavoidable, due to the large consumption of iron ore resources and a general Cr and Ni shortage. The use of scraps determines in the manufactured alloys the presence of more noble elements such as Cu, As and Sn because they cannot easily be removed. The advantage is that elements such as Sn give a significant improvement in corrosion resistance of low-Cr (14 wt.%) ferritic SSs in less severe environments, compared to the common 18 wt.%-SSs. Alloys such as those without Ni, Mo, Cu, but with a 16 wt.% of Cr and 0.3 wt.% of Sn gave the same corrosion resistance as 304 SSs, saving 40% of the total Ni and Cr consumption on the production of SSs and so reducing the external market dependence in industrial processing.

In terms of corrosion resistance, the presence of Cr in SSs, in a sufficient amount, guarantees their strong corrosion resistance, oxidation resistance and/or heat resistance. Typically, it ranges from 10.5 wt.% to 30 wt.% [14,15]. More specifically, reacting with oxygen from the atmosphere, Cr determines the passivation of SSs, producing an adherent, insoluble, ultrathin (1–2 nm) film that protects the alloy against corrosion, in particular under wet conditions, where acidic species and/or chlorides-contaminated environments are present. For stable surface passivation, the content of Cr should be at least 10.5 wt.% [8,14,16,17,18]. Furthermore, the Cr present in the bulk of SSs guarantees self-healing properties, which enable restoring their corrosion resistance by forming a new passive Cr-based oxide layer in the presence of oxygen in the event of surface mechanical scratches or damage [14,17]. The formation of a Cr_2_O_3_ thin layer on SSs by Cr gives also the necessary protection in high-temperature applications [14].

A partial substitution with limited options [9] and/or a reduction of Cr in SSs steel alloys could be possible, maintaining the corrosion properties of these alloys [19]. It seems that there is not a chance to completely substitute this element within the corrosion performance of SSs [8]; it is a utopia (or “a dream” [16]) and probably remains an old question [20] without a response, in particular in those applications, such as severe aqueous environments where the high corrosion resistance properties of SSs are needed. Several U.S. researchers have faced up to this question during the 1980s and 1990s because it has been considered a fundamental issue due to the possible import vulnerability of their country in supplying strategic materials [21], such as the situation previously discussed, coming from EU reports on analyzing CRMs.

Dealing with the corrosion performances of metallic materials, the difference between the resistance to oxidation in high-temperature applications (“dry corrosion”) and the resistance to corrosive electrolytic aqueous environments (“wet corrosion”) has to be examined separately on searching for alternative elements to Cr in SSs, at least for maintaining their corrosion properties. In the obtainment of new SSs, they have to be comparable in terms of corrosion resistance, at least to the ones typically used of the 300-series, as AISI 304 and AISI 316 passing from a content of Cr 18 wt.% to 8–12 wt.% [16,21,22], reduce the consumption of Cr to one-third in this way. This seems to be feasible in less demanding exposure conditions, but sometimes there are some possibilities also in severely corrosive environments. In any case, this represents a hard challenge especially in wet electrochemical corrosion, to which this review is mainly addressed.

### 2.1. Stainless Steel under “Dry Corrosion” Conditions

The possible elements which are candidates to partially substitute Cr, include especially Al and/or Si in different content combinations. However, other elements are also taken into consideration. In applications where corrosion at high temperatures occurs [22], Cr can be replaced by the aforementioned elements. The performances of the common SSs, AISI 304 and AISI 410 (a ferritic 12% low-chromium steel), have been compared to (8-10)Cr-(10-14)Ni-(0-8.5)Si-(0-4)Al (wt.%) alloys, which gave better results. More specifically, alloys containing 8% or 10% of Cr and 5% of Si were tested in hot conditions at 700 and 800 °C, and an improvement of corrosion resistance and mechanical properties was achieved.

Other authors highlighted the specific efficiency of Si and Al in enhancing the high-temperature oxidation properties of SSs [23,24,25], reporting, in particular, the significant role of the only Si [24] additions and the additions of Si together with Al at 700 °C. Increasing the temperature to 800 °C, Al considerably reduces the resistance to oxidation of these newly tested alloys, due to the higher affinity of Al for oxygen compared to Si, promoted by the increase in temperature, which favors the formation of aluminum oxide with respect to the more protective SiO_2_ film, present in the alloys containing only Si. In other studies [26], the efficiency of Al in improving high-temperature performances (up to 1375 °C) has been shown in specific Fe–Cr alloys with a high-Cr content (40 wt.%), where a partial substitution of this element by Al brought the formation of a protective α-Al_2_O_3_ layer. Together with Al, microalloying with Ru (0.2 wt.%) further enhanced the corrosion properties of Fe–Cr–Al alloys. This replacement of Cr by Al could be considered a useful way of reducing the Cr consumption, but with the limitation not going under 20 wt.%, which impairs the oxidation resistance and the durability of these alloys.

A protective layer of Al_2_O_3_ resulted in the key factor also for Fe–Cr–Al–Mo oxide dispersion strengthening (ODS) alloy (commercially known as KANTHAL©, developed by Sandvik) for corrosion resistance at high temperatures, up to 1250 °C, where chromia-forming Ni-based alloys containing more Cr can reach lower application temperatures of 1000–1100 °C [27]. At high temperatures, these last materials suffer from accelerated oxidation and softening of grain boundaries. ODS guarantees the high-temperature strength of Fe–Cr–Al–Mo alloys.

Mo, Cu, V, N and Ni have been proposed for partial reduction of Cr content from 18 to 9 wt.% [16,21], where Ni is necessary (up to 24 wt.%) for maintaining the austenitic microstructure, with the consequence of increased material costs [16] due to the fluctuation of Ni prices [28]. The complete substitution of Cr has been tried by examining Fe–Al–Mo and Fe–Mn–Al alloys obtaining lower hot corrosion performances compared to those of the 300-series [21,29]. Finally, Fe-31Mn-7.5Al-1.3Si-0.9C (wt.%), an austenitic SS, has been used to obtain steel wires for high-temperature applications. It gave similar results as AISI 304 for temperatures up to 700 °C, but the properties were inferior at 800 °C [30].

### 2.2. Stainless Steel under “Wet Corrosion” Conditions

Passing to the review of wet corrosion performances of SSs, the replacement of Cr, even partially, is possible but mainly in less demanding corrosive environments [16,21]. It has been demonstrated that corrosion resistance increases with the increase in Cr content in SSs, both in acidic and strongly chloride-contaminated environments [16,31], as expected.

To reduce Cr in the SSs, the possible strategies should take into account that the content of this element has never been lower than 12 wt.% to find comparable performances to AISI 304, as reported in a very old paper [32]. An amount of 2–3 wt.% of Si or Mo in these austenitic SSs (Ni 9 wt.%) improved the corrosion performances both in H_2_SO_4_ 1N and in NaCl 1N. The results show the high tendency of passivation for active–passive metals, such as SSs, in the acidic environment due to the reduction of critical passivation current density, and the decrease in pitting susceptibility in NaCl 1N due to an increase in pitting potential. It has also been reported that the addition of Al has not exceeded 1 wt.%, because it determines a decrease in the pitting potential. The increase in this element is beneficial for high temperature corrosion. The optimized alloy composition was 10Ni-1.5Si-1Al-2Mo (wt.%) for achieving the same corrosion performances as 304 SS in the above-mentioned solutions, which cannot be considered as extremely severe exposure conditions. On the contrary, in more recent papers, SSs with a lower amount of Cr than 12 wt.% have been investigated to study their corrosion performances. Austenitic 9Cr-12Ni based SSs, with the addition of Mo (2–5 wt.%), V (0–2 wt.%) and Cu (0–2 wt.%), were again compared to 304 SS to substitute it [33]. They were examined in acidic environments (H_2_SO_4_, citric acid and H_3_PO_4_), at different concentrations and temperatures and in NaCl at low (0.016 wt.%) and high (3.56 wt.%) concentrations. In an acidic environment (general corrosion), 2 wt.% Mo in the alloy improved the performances of these materials with respect to 304 SS; in a neutral NaCl environment, the difference between all examined materials is unimportant. Another example of low-Cr austenitic SSs is Fe-8Cr-16Ni-5Si-1Cu, with and without 1 wt.% of Mo [23]. They were tested in different acidic solutions and compared again to the corrosion performances of AISI 304. Given the low percentage of Cr in these alloys, Si and Cu were added to maintain their high wet-corrosion resistance. These elements are ferrite stabilizers, and therefore 16 wt.% of Ni was necessary to maintain the austenitic structure, corresponding to a higher amount with respect to that present in 304 SS (10 wt.%). Good results have been obtained because similar corrosion behavior was obtained and the new alloys represent an interesting material to reduce Cr consumption, even if the necessary increase in Ni does not correspond to a good commercial solution.

Concerning the effect of Al, a relatively recent study has been made on the effect of this element and/or Si on ferritic SSs, with chromium ranging between 10 and 18 wt.% [34]. With not more than 12 wt.% of Cr, in an NaCl 3.5 wt.% solution, the presence of 3 wt.% of Al increased the pitting potential, which was further increased by adding at least 1 wt.% of Si, even if it impaired the repassivation properties of the examined SSs, which cannot be neglected. In H_2_SO_4_ 0.05 M (active corrosion), the beneficial effect of 3 wt.% of Al and 3 wt.% of Si, added separately to the analyzed SSs, has been observed in terms of the reduction of passive current density, up to a weight fraction of Cr equal to 16 for the former and independently from Cr concentration for the latter. These authors showed the beneficial effect of Al, by a percentage higher than 1 wt.%, both in an acidic and neutral environment contrary to the findings of the previously cited authors [23], because it is significantly related to the Cr content in the 10–18 wt.% range, whereas these last authors examined alloys with a lower Cr content (8 wt.%). Furthermore, these considerably different results could also be attributed to the different types of tested SSs: austenitic [23] vs. ferric ones in [34]. The presence of Ni being the difference between them, it can also be considered that this element plays a role in the corrosion resistance of austenitic SSs, as reported in the literature on retarding their corrosion, at least in mineral acids [35,36,37].

Considering again the role of Al, it is beneficial in the protection of new austenitic SSs (Ni 15–18 wt.%) with low-Cr (10–11 wt.%) and high-Al (around 5 wt.%) contents, as demonstrated in severe electrolytic environments such as concentrated HNO_3_, up to the azeotropic point (65–67 wt.%). These acidic solutions are used, for example, in the production of acrylonitrile textile fibers and nuclear fuel reprocessing [38]. Different alloys were tested by weight loss measurements through the Huey test (ASTM A262, practice C), consisting of their immersion for five cycles of 48 h in boiling HNO_3_ at the concentration of 65 wt.%. In more detail, the alloy Fe-18.27Ni-10.6Cr-4.72Al-4.98Mn-0.36Si-0.05C (wt.%; Si must not exceed 0.5 wt.%), having an almost complete austenitic matrix, showed weight loss comparable to 304 SSs due to the formation of a protective layer of aluminum oxide, determining in this way the saving of about 7% of Cr.

The improvement of corrosion resistance of SSs containing Si and low Cr amounts (8–13 wt.%) has been found by other authors [39] testing in different concentrations of H_2_SO_4_ solutions (0.1–1 N), also in the presence of sulfates, when the sum of Si and Cr concentration is above an atomic fraction of 14%. When this sum was above 15%, the resistance to pitting corrosion was increased in solutions with 0.2 g/dm^3^ of NaCl. The benefit of Si has not been well understood, but it seemed related to the formation of fayalite (Fe_2_SiO_4_), which could stabilize the protective film of the studied alloys.

Ferritic SSs such as 430 (16.5 wt.%) and 409 (11.5 wt.%), which are low-Cr and Mo-free materials, considered critical alloying elements in terms of costs, have been studied as a potential substitution of the more expensive 316L SS in desalination plants for application in less aggressive conditions [40].

Some authors studied the peculiar protective properties of the passive layer of ferritic SSs such as Fe-17Cr, Fe-12Cr-5Al and Fe-13.5Cr-3.5Si (wt.%), with a small addition of Mo, by the electrochemical cathodic reduction method, which can be correlated to their localized corrosion resistance [41]. If these materials give similar results by means of this method, a guarantee of similar localized corrosion properties could be supplied, and then there is the possibility to have an investigation technique for designing alloys with lesser amounts of Cr, replaced by Si and/or Al. Dealing with these last two elements, it must be considered that they are ferrite phase stabilizers, therefore to promote the formation of an austenitic phase for the obtainment of SSs with good mechanical properties, elements such as Mn and Ni are necessary. Ni (10 wt.%) is preferred in place of Mn [19,32].

Concerning high-Cr alloys, Fe-40Cr (wt.%), considered in Section 2.1 for the heat oxidation resistance, where Al has been found a good element to partially replace Cr, this cannot be completely stated in wet corrosion conditions [26]. Fe-35Cr-5Al alloy gave a similar anodic behavior of the Fe-40Cr alloy in a de-aerated 10% H_2_SO_4_ solution at 25 °C, whereas a considerably lower pitting resistance has been found for the former with respect to the latter in NaCl 3.5 wt.%. Even in this case, a Ru (0.2 wt.%) micro-addition improved the wet corrosion resistance. Including noble metals such as platinum-group metals (PGMs) in these alloys promotes self-passivation properties in acidic solutions: they determine the increase in hydrogen exchange current, bringing these alloys towards passive conditions. It was observed that the amount of Cr can be reduced, increasing the percentage of noble elements. Ru micro-additions produced also the increase in localized corrosion resistance in an NaCl solution for the alloys where Al replaced Cr.

Even within duplex SSs, Fe–Cr–Mn–Al–Ni systems with low Cr (15 wt.%) and low Ni (2 wt.%) were studied for substituting the most common Fe–Cr–Ni duplex systems. These new duplex SSs make necessary the additions of Al (1–3 wt.%), which stabilizes the ferritic phase and Mn (8–12 wt.%), which stabilizes the austenitic phase [42]. Good pitting corrosion resistance has been obtained in NaCl 3.5% at 25 °C, which initiates in the austenitic phase due to the presence of manganese sulfide formed by Mn present in this phase.

The complete substitution of Cr, as already observed, is impossible for wet corrosion and experimentally confirmed by trying to use Fe–Al–Mn (“poor man’s SSs”) alloys in place of Fe–Cr–Ni alloys, without success [43,44].

Recently, some authors proposed new interesting lightweight SSs (6.3–6.5 g/cm^3^), which have a reduced density with respect to the well-known Fe–Cr–Ni and Fe–Cr SSs (7–8 g/cm^3^), maintaining their corrosion and mechanical performances [45]. They are based on the system Fe-(20-30)Mn-(11.5-12)Al-1.5C-5Cr (wt.%), where Ni is absent. Therefore, these Fe–Mn–Al–C systems determine also a decrease in the material costs and save a significant amount of Cr (about 5 wt.%), where for the most common Fe–Cr–Ni systems a minimum amount of 10.5 wt.% is requested, as reported above. Al determines the decrease in the alloy density and the possibility of forming the aluminum oxide protective layer in combination with the 5 wt.% of Cr; Al being a ferrite stabilizer element, in the absence of the expensive Ni, Mn and C austenite stabilizers, must be increased up to 30 and 1.5 wt.%, respectively. It is well-known that high-C SSs (e.g., 1.5 wt.%) promote the formation of Cr carbides, which determine their intergranular corrosion. In these new alloys, this risk has been eliminated by the strong decrease in Cr content with respect to that present in the common ferritic and austenitic SSs, avoiding the formation of carbides at the grain boundaries. These compounds form in fine ordered forms in the austenitic grain matrix, determining thus the strengthening and the ductility of these new SSs. In terms of corrosion, new leaps forward have to be made, because the pitting corrosion resistance in NaCl 3.5 wt.% solution is comparable only to that of ferritic SSs (e.g, 430) and not to that of austenitic SSs (e.g., 316).

In the previously mentioned alloys, obtained by recycling SSs scraps which contain Sn present as oxide with Cr oxide thus stabilizing the passive film, an improvement in corrosion protection and in the self-healing characteristics of the manufactured alloys has been shown [13]. On the contrary, it is necessary to pay attention to the segregation of Sn at the grain boundaries, which could determine the embrittlement of the metallic materials.

From the analysis made so far, considering the modification of bulk alloys, there are really effective solutions for replacing and/or reducing Cr consumption but in less demanding environments, especially in terms of corrosion resistance in wet conditions, apart from the very singular case of exposure in boiling HNO_3_ azeotropic solution. Therefore, it is clear that it is a huge challenge to develop SSs with suitably adjusted composition able to face up to corrosion in severe (“extreme”) environments because positive results have been obtained in less-demanding ones. Taking into consideration the elements reported in this review, Al, Si and Mo play an important role in this progress of the research and technological applications. In this context, it must be taken into account that Al and Mo are outside the CRMs criticality area, while Si is inside it in the 2014 report on CRMs [9] but is becoming closer to the SR border in 2017 [10] and 2020 [5] reports. Considering also the mechanical properties and addressing the attention on the ductility of the new possible alloys, Mn (slightly outside the CRMs criticality area in 2020 [5]) has not been excluded from this investigation, if an austenitic matrix has to be maintained. On one hand, the use of Ni is preferable compared to Mn not only in terms of an austenitic phase stabilizer, but also due to its contribution to increasing the corrosion resistance of SSs, especially in acidic environments. On the other hand, nickel’s high and fluctuating price cannot be neglected, consequently influencing that of SSs; thus, new strategies in the development of new alloys containing Mn are proposed [46] and could be promoted.

A promising strategy in the direction of saving Cr and maintaining the corrosion performances of the most common SSs is that which considers the development of new Fe-based alloys. They have a low-Cr content in the bulk (e.g., low-alloyed common steels or some ferritic SSs), whereas the surface has a Cr-rich coating [21] and/or other protective coating solutions [47], containing in the bulk Mn [46] in place of Ni. Through laser and plasma technologies or ion implantation, a surface alloying of Cr or Cr, C, N, etc.-rich surfaces can be obtained as reported by the following examples.

An AISI 410 martensitic SS (12.2 wt.% Cr) subjected to plasma nitriding [47] at 500 °C, where iron nitrides were formed in place of the CrN, showed increased corrosion properties both in 1 vol.% HCl and NaCl 3.5 wt.%. The formation of CrN typically requires high operating temperatures and if they are higher than 550 °C, the depletion of Cr in the grain matrices occurs leading to a decrease in pitting corrosion resistance. Therefore, the temperature has to be lowered, and to make it possible, the process can be carried out in molten salt baths, such as a KNO_3_ bath at 450 °C, to obtain less expensive Cr (13.5 wt.%)-Mn (9.1 wt.%) austenitic SSs having a nitride protective layer, with respect to untreated Cr–Ni ones. The formation on this SS alloy of a 3 µm thick nitride layer leads to a higher corrosion resistance than that of untreated 304 L, comparable to untreated 316 L in NaCl 3.5 wt.%, considering electrochemical impedance spectroscopy and potentiodynamic tests [46]. Therefore, this technology not only allows the reduction in Cr content in the alloys but also promotes the use of Mn in place of Ni to produce austenitic SSs for many commercial and industrial purposes, which cannot be considered possible based on previously examined strategies.

In recent years, the high-Cr ferritic ODS steels have gained much research interest, even if they have less workability than low-Cr (8–9 wt.%) ferritic/martensitic SSs, which in turn do not reach the corrosion resistance of the first ones [48]. For increasing the corrosion performances of low-Cr alloys, they were coated with a thickness of around 10 µm, using mechanically alloyed powders having a composition of 304 or 430 SSs. Similar results can be obtained by depositing similar materials on less noble substrates through powder spraying technology [49]. The corrosion resistance is improved at the same or a higher level than 316L, when a Fe-25Cr-10Mn-(<2)B-(<2)C (wt.%) alloy coating is deposited; in addition, this alloy permits achieving also a significant increase in abrasive resistance of the material.

Manufacturing “multi-layered steel materials” also known as Spatially Optimized Diffusion Alloy (SODA) materials are an interesting technology for saving chromium [50]. The bulk of these materials contain small amounts of this element (around 3 wt.%), which increase going toward the surface (more than 20 wt.%), for a thickness of about 70 µm, in contact with the corrosive environments, therefore giving them the necessary corrosion resistance. In addition, the low-Cr content in the bulk makes the manufacturing processing of the SODA materials easy due to the absence of hardening promoted by this element without significant modifications of the industrial equipment, obtaining coatings ranging from 20 to 200 µm in thickness. If the core material is a 1.5 mm thick steel sheet and the coating is 70 µm thick, on each side the average Cr concentration is 1.9 wt.%, saving in this way a large amount of the element if compared to that present in the common SSs, ranging from 10 to 20 wt.%. In particular, SODA materials do not show coating cracks and/or loss of adhesion after 180° bending tests, which is a fundamental aspect for preventing the contemporary contact of the less noble substrate together with the more noble coating in corrosive environments.

Finally, for improving corrosion performances of SSs, there is also the possibility of submitting them to acidic-based processes [14,15,17,51,52]: namely, passivation treatments, even when their Cr content is equal to 18 wt.%. Passivation treatments increase their durability and so they can be considered another strategy for saving Cr. Typically, the passivating solutions are based on nitric acid or on a mix of this acid and hydrofluoric acid (having also pickling properties). In light of “green chemistry” and “life cycle assessment”, some authors studied the use of citric acid at different concentrations and temperatures, having less environmental and human health impact concerning nitric acid [52]. This study has been made on different grade SSs to obtain by citric acid treatments similar corrosion performances given by nitric acid passivation, but unfortunately the authors of this work did not report any quantitative results coming from corrosion tests.

### 2.3. Cr as CRM in Stainless Steel—Summary

From this review, the conclusion is that the complete substitution of chromium in SSs is impossible, or at least difficult, in terms of corrosion resistance, in particular when new bulk alloys have to be manufactured. Within the heat oxidation resistance of SSs, Al and/or Si are the more promising elements for replacing Cr compared to the applications on wet corrosion, in particular in severe exposure conditions. However, within wet corrosion, new adjustments of SS compositions are recently taking place with interesting results on comparing the characteristics of the newly manufactured alloys to those of the 300-series alloys. When it is technologically feasible, improving the corrosion resistance limitedly to a surface layer/coating of low-bulk-Cr alloys, up to those with the practical absence of this element, represents a very promising strategy for decreasing the consumption of Cr. The manufacturing of these new materials not only maintains corrosion resistance of typical SSs, but also guarantees good mechanical and wear performances. On the other hand, going toward this direction, especially in terms of corrosion resistance, it has to be taken into account that the possible noble coating or improved passivation layer must not contain defects (e.g., cracks) during the industrial processing of the materials and/or during field applications. In the presence of defects, different noble materials are contemporarily in contact with corrosive environments determining their extremely unfavorable galvanic coupling, which is responsible for selective or localized penetrating corrosion, determining the failure of metallic components under service conditions.

## 3. Substitution of Critical Raw Materials in Tool Steel

Tool steel’s desired properties, such as high hardness, wear-resistance and also thermal stability (in the case of high-speed steel and hot work tool steel), are reached by the combination of high carbon levels, alloying and appropriate heat treatment. The alloying elements are mainly carbide-formers (tungsten, vanadium, molybdenum and chromium). In high-speed steel, cobalt is also used to improve the thermal stability of the tools. Among the mentioned alloying elements, tungsten, vanadium and cobalt are nowadays listed as critical raw materials [8]. In the 2014 CRM list, chromium was also included [9], and this element has still to be considered strategic, as discussed above. From the economic and sustainability points of view, substitution of the above-mentioned elements is of high importance. A certain level of substitution can be achieved based on a deep knowledge of the behavior of these elements during steel processing, heat treatment and service. As mentioned above, the role of tungsten, vanadium and chromium are key elements for the formation of hard carbides.

### 3.1. Rational Approach to the Substitution of CRMs in Tool Steel

Since the microstructures of steels evolve during processing and determine the properties of the final product, it is necessary to be able to describe these changes. Phase transformations and other microstructural changes such as recrystallization, grain growth and recovery effects may occur when the steels are subjected to specific heat treatments. The evolving microstructure can be measured using in situ X-ray diffraction (XRD). The experimental data are evaluated by the Rietveld method, where further techniques, e.g., the double-Voigt peak broadening model, are used to refine the model parameters [53]. Based on this further refinement, the transient phase fractions and dislocation densities during a complex thermal regime can be obtained [54]. In addition, precipitation of, e.g., secondary hardening carbides [55] or intermetallic phases can be monitored directly or indirectly by in situ X-ray diffraction [56]. Recently, it has been shown that even processes yielding low signal-to-noise ratio diffraction patterns can be analyzed by a parameter refinement with a global optimizer using a Bayesian approach with a Markov Chain Monte Carlo algorithm instead of the standard Levenberg–Marquardt local optimization technique [57]. Therefore, it becomes possible to monitor transient microstructures during comparatively fast heating and cooling processes using standard laboratory in situ X-ray diffraction equipment.

High-speed steels outperform other steels due to their excellent cutting performance and hot properties. High Temperature–X-Ray Diffraction (HT-XRD) investigations have the potential of helping design an optimized heat treatment for improving the mechanical properties of the steel grades and thereby coming into the possibility of avoiding CRMs. The relative importance of the microstructural contributions to the hot properties of high-speed steels (matrix strengthening and strengthening due to undissolved carbides) have been investigated in a pioneering work [58] with the aim of monitoring these changes directly by XRD. It is a challenging task to design, e.g., Co-free maraging and high-speed steels while maintaining the mechanical properties of the Co-containing grades. Li et al. [59] obtained ultrahigh strength steel by a high density of nanoscale precipitates with a low lattice misfit. Fathy et al. [60] determined the optimized heat treatment for several low-nickel cobalt-free maraging sheets of steel. All these investigations can be supported by in-situ X-ray diffraction experiments.

**Figure 2:**
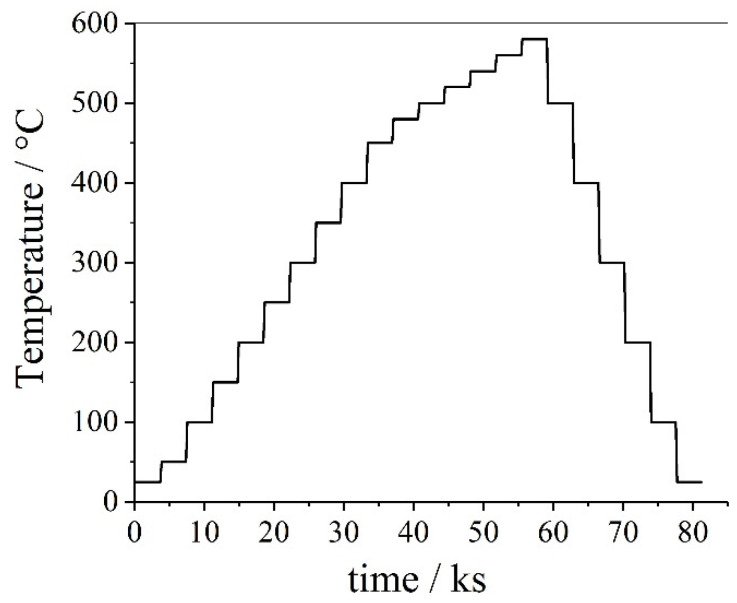
The time–temperature curve for the in situ heat treatment during the X-ray diffraction (XRD) measurements.

As an example, the microstructural changes during heat treatment of a powder metallurgically produced high-speed steel S290 PM containing a considerable amount of tungsten, and cobalt was investigated using HT–XRD. The composition of the steel grade considered is provided in Table 2. Heat treatment was subsequently applied to high-speed steel in order to investigate the influence of various temperatures on the evolving microstructure (see Figure 2).

**Figure 3:**
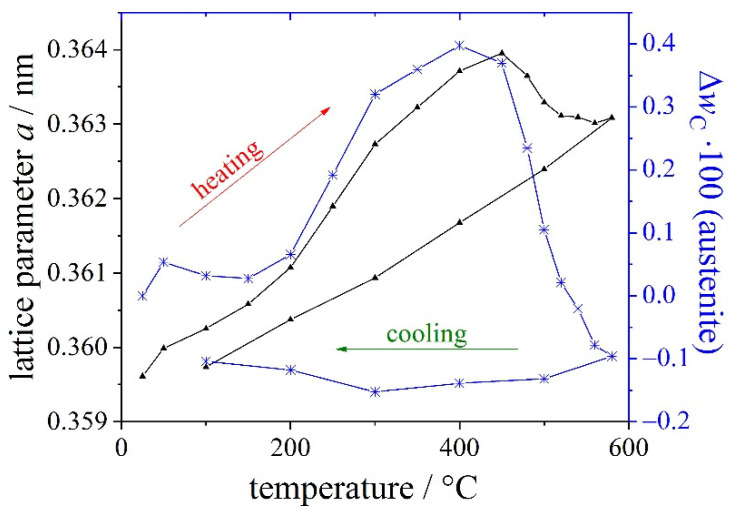
Lattice parameter *a* of austenite (black curve with small triangles) and change in carbon mass fraction Δ*w*_C_ (blue curve with stars) versus temperature during a heating/cooling cycle.

The partitioning of carbon plays a key role in the microstructure and the properties of this high-speed steel. During heating, there is a change in the lattice parameter due to the change of the carbon content in the steel in addition to thermal expansion. Thereby the change in the amount of carbon in austenite is calculated [61]. The evolution of the lattice parameter *a* and the derived change of mass fraction of carbon in austenite in percent (Δ*w*_C_ 100 in austenite) are plotted in Figure 3. Carbon diffuses from martensite into the austenite during heating between 200 °C and 400 °C. Carbon accumulates in austenite during this stage. Nano-carbides are formed at temperatures higher than 400 °C, and thus the mass fraction of carbon decreases in austenite. After tempering during cooling, the lattice parameter a of austenite follows the temperature-dependent thermal expansion curve; i.e., the mass fraction of carbon in austenite remains almost constant.

**Figure 4:**
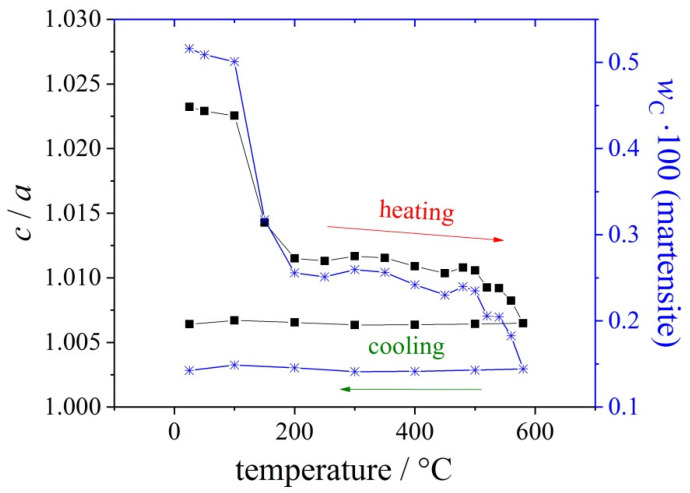
Tetragonality *c*/*a* of martensite (black curve with squares) and carbon mass fraction in martensite (blue curve with squares) versus temperature during a heating/cooling cycle.

The mass fraction of carbon in martensite is estimated by measuring the tetragonality; i.e., the *c*/*a*— ratio of the tetragonally distorted unit cell of martensite. It is worth mentioning that the change of the lattice parameter in austenite allows estimating the change of the mass fraction of carbon in austenite only, whereas the *c*/*a*—ratio is directly proportional to the absolute mass fraction of dissolved carbon in martensite. Thus, the carbon mass fraction in martensite by percent (Δ*w*_C_ 100 in martensite) is calculated from the tetragonality *c*/*a*. The evolution of the *c*/*a* ratio and carbon mass fraction in martensite is plotted versus temperature for a heating/cooling cycle (Figure 4).

After quenching, a large amount of carbon is dissolved in martensite; i.e., the *c*/*a*—ratio is high. During heating, carbon atoms up to 200 °C can leave martensite, and thereby the *c*/*a*—ratio is strongly reduced. Carbon-containing clusters are likely to occur before carbides can precipitate during this stage of the heating process. These clusters occur at low temperatures in martensite and to a smaller extent also in austenite. This phenomenon is described in [62]. The diffusivities of the sluggishly diffusing substitutional components are orders of magnitude higher in martensite than in austenite at the same temperature. The further drop of the *c*/*a*—ratio at higher temperatures is due to precipitation of nano-carbides or secondary hardening carbides.

**Figure 5:**
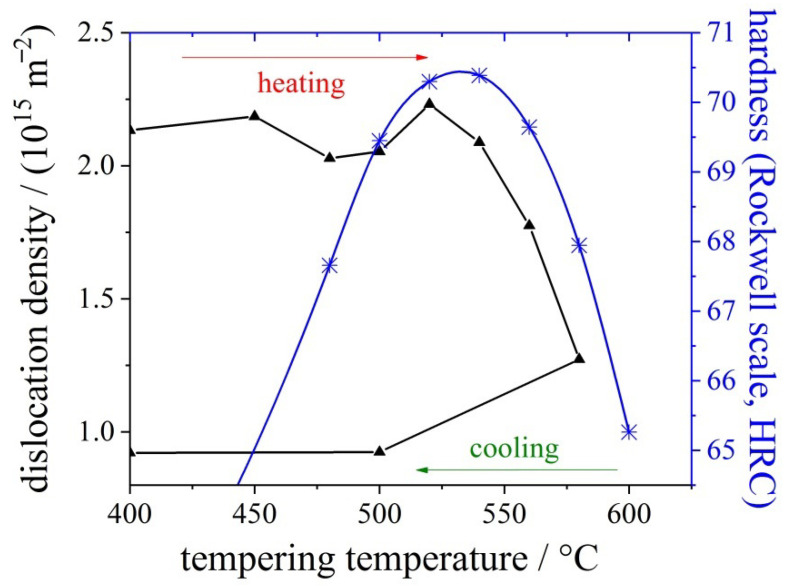
In situ dislocation density in martensite derived from the microstrain (black curve with triangles) versus temperature during a heating/cooling cycle and ex situ Rockwell hardness in martensite (blue curve with stars).

These results can be compared to the temperature-dependent hardness (Figure 5). The maximum in the hardness curve at approximately 540 °C can be explained by precipitation of the above-mentioned nano-carbides measured by the decrease in lattice parameter and by a drop of the dislocation density in martensite.

It has been demonstrated in this work that HT-XRD is a powerful tool for the in situ evaluation of process conditions such as tempering. Based on the experimental results, it is possible to identify relations between the evolving microstructure and mechanical properties. The evolution of the microstructure during the tempering process of the S290 PM high-speed steel was revealed. It was indirectly shown that diffusion of carbon from martensite to austenite during heating and later the precipitation of nano-carbides are processes required to provide excellent mechanical properties to the S290 PM steel. This steel grade contains a high number of CRMs. The next task is thus to design steels where similar microstructural changes are expected with steels containing a reduced number of CRMs or different composition and to compare the microstructural evolution during heat treatment of this steel with steel grades containing a significantly reduced amount of W and Co in future work.

The substitution of alloying elements has been carried out already in 1950–90, when the tool steels alloyed just by chromium and high carbon level have been developed over the world. The reason was the unavailability of tungsten. However, such steels do not reach the properties of the above-described high-speed steel. Due to limited thermal stability, these alloys could be used just as cold-work tool steels. At the beginning of the 21st century, there were also trials in substituting vanadium partially with niobium [63,64,65,66]. These elements form the same type of carbide (MC), while NbC is harder than VC. On the other hand, niobium carbide tends to coarsen during metallurgical processing. Even though a suitable manufacturing route (rapid solidification powder metallurgy) [66] and appropriate surface treatment [67] were developed, it did not lead to a high success due to the increasing use of niobium and its low availability, which led to its listing as a CRM. The other possibility for substituting vanadium partially is titanium [63]. The manufacturing problems connected with the production of such steel are comparable with those for the Nb-alloyed one, and hence they could be solved by the use of powder metallurgy.

### 3.2. Hard Coatings of Tools as a Means of Saving CRMs

Tool lifetime and resistance to high working temperatures as critical requirements for producing cost-effective tool steels [68,69] can also be achieved by producing cheaper tool steel, compared to the high speed steel (HSS), by applying a surface treatment such as plasma nitriding, for example, and/or by depositing a hard coating. Moreover, this practice has also the advantage of improving the tribological properties of the coated tool steel [70]. Nevertheless, more importantly, the use of coating on tool steels is likely to go in the direction of minimizing the use of the critical raw elements (CRE) such as W and Co that are typically added to the HSS. To produce high-quality parts, a cutting tool must have high hardness and strength at high working temperatures, sufficiently high toughness, and wear resistance over all the possible working temperatures, which are from room to the maximum working temperature. To reach this level of mechanical properties, the cutting surfaces of tool steel can be coated, and thence modified by advanced physical vapor deposition (PVD) technologies. PVD is recognized as an environmentally friendly technique since it does not require the use of harmful chemical agents and gases. In this context, several possible elements can be used to effectively coat tool steel to meet the needed requirements and to reach the typical mechanical properties of HSS. These include binary compounds, such as nitrides (TiN, CrN, NbN) which can be produced in the form of multilayers (i.e., alternate nanometric layers of CrN and NbN), or ternary and even four- or five-element compounds. As per the ternary coatings, a promising one is surely the multilayer titanium boron-nitride, TiBN [70,71,72]. On the other hand, several four- and five-element coatings are known to effectively harden tool steel surfaces; these include Al-Ti-Cr nitrides, Al-Ti-Cr carbo-nitrides or Al-Ti nitrides added with another metallic element (Si, Y, …) [68,73,74,75]. Concerning the simplest binary coatings, their main drawback compared with ternary or quaternary coatings is their limited oxidation resistance, with a typical allowable working temperature of within 500 °C. On the other hand, maximum hardness values of 3.5 to 5 GPa are likely to be reached in several nitride multilayer binary systems, most of them up to 1000 °C (e.g., TiN/AlN [76], TiN/NbN [77], TiN/VN [78], TiN/CrN [79], AlN/CrN [80]). 

**Figure 6:**
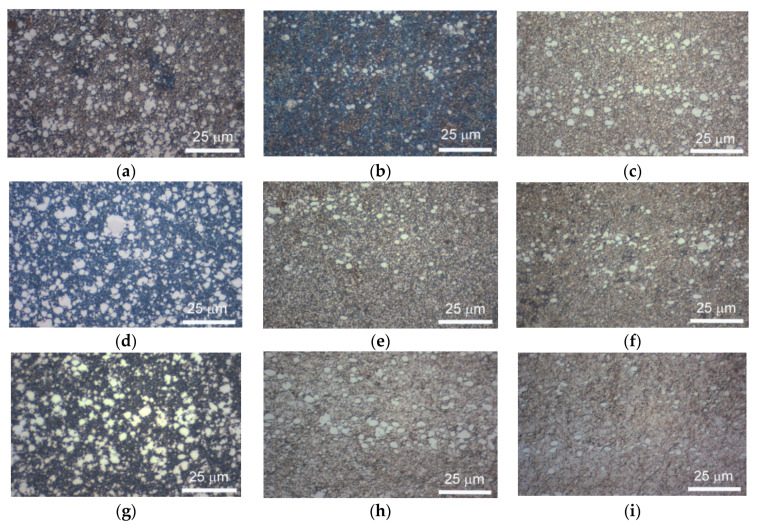
OM (optical microscopy) of the TiAl_1-x_Cr_x_N coating after cycle I (**a**,**d**,**g**); cycle II, (**b**,**e**,**h**); and continuous exposition (24 h) (**c**,**f**,**i**) at maximum testing temperature of 800 °C (**a**–**c**); 1173 K, (**d**–**f**); and 1000 °C, (**g**–**i**).

**Figure 7:**
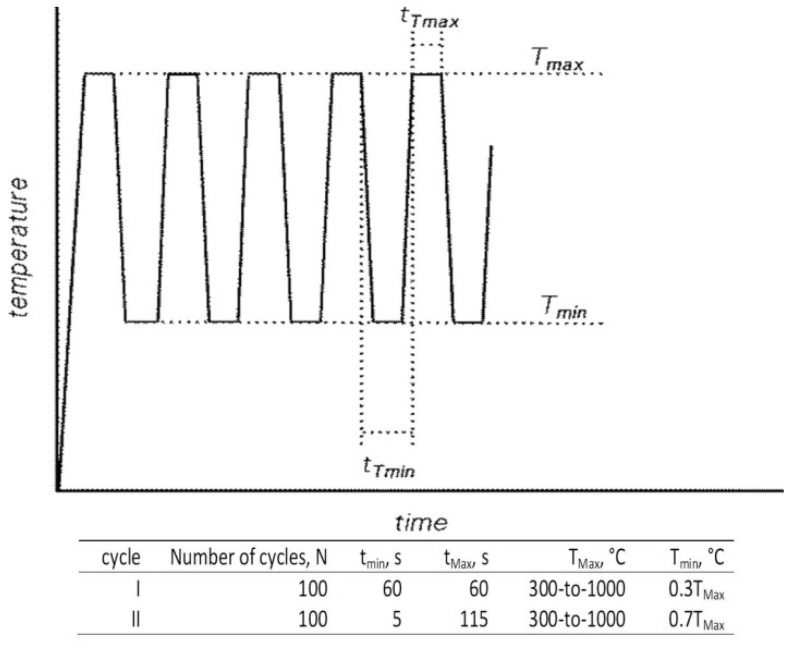
Schematic representation of the two thermal cycles to which the TiAl_1-x_Cr_x_N + tool steel was subjected to determine the minimum working temperature by which surface oxidation started to form on the coating.

Exceptional wear resistance and hardness can be reached by quaternary advanced coatings, such as the multilayer TiAl1-xCrxN, which mainly consist of a very high oxidation resistance up to ~1100 °C and high hardness, typically one-order of magnitude greater than the binary multilayered coatings, as H was reported to be up to 30 to 50 GPa [74]. The required stability at high working temperatures also includes grain size stability, as in these coatings phase boundaries act as an effective barrier against defect propagation. In this regard, the multilayer nature of complex coatings is known to ensure a sufficiently high level of grain and layer stability against temperature cycling [74,75,76,77,78,79,80]. In multilayer TiAl_1-x_CrxN, (Ti,Al)N nanocrystals form, and their typical size is ~5 nm; these are evenly distributed within the coating. The exceptional high wear resistance of the multilayer TiAl1-xCrxN coating during high-speed machining is ensured by the formation of a protective alumina film on the cutting tool surface [81,82]. Experimental studies on the TiAl1-xCrxN quaternary hard coating on tool steels revealed optimum thermal cycling endurances, quite high hardness levels for wide working temperature ranges and good wear resistance performances. These significant mechanical properties were inferred by nanoindentation, wear and thermal cycling tests; the main results are summarized and outlined hereafter. Experimental methods and procedures can be found elsewhere in previously published papers by one of the present authors (Cabibbo et al.) [83,84,85,86,87]; thence, no changes in the surface roughness were observed up to 800 °C. The external surface of quaternary TiAl1-xCrxN (with x varying from 0.3 to 0.7) started to form scattered oxide spots whenever continuously exposed to a temperature of 800 °C. Surfaces further deteriorated as the exposure temperature rose to 1000 °C. Thermal cycling tests revealed the same oxidation behavior which started by thermal exposition from 800 °C of a maximum temperature excursion. Figure 6 shows optical microscopy (OM) micrographs, which correspond to thermal cycles simulating working conditions to which the stem valves, dies or cutting-edge tools are typically subjected. The simulated thermal cycles were the ones reported in Figure 7.

**Figure 8:**
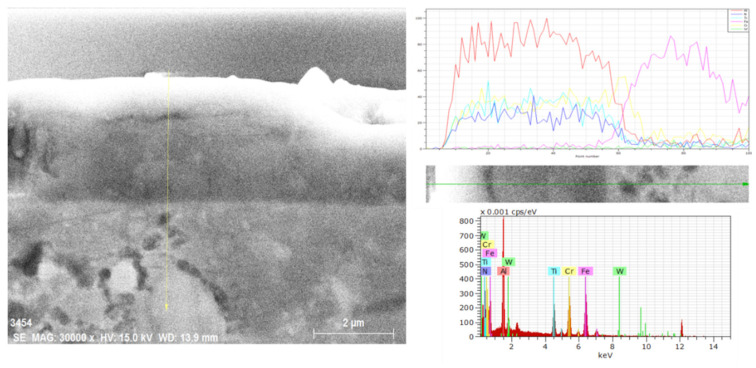
Representative FEG-SEM + EDS line scan analysis of the coating profile at 800 °C.

It resulted that oxidation phenomena did not significantly depend on the specific thermal cycle. Indeed, by exposing the AlTiCr_x_N_1-x_ coating to high temperatures, starting from 800 °C, segregation of titanium and chromium atoms toward the substrate occurred. This thermodynamic-activated atomic segregation mechanism does involve the diffusion of aluminum atoms to the surface. This, in turn, forms a very thin aluminum oxide top layer. As a matter of fact, with regards to this structural modification induced by cycling temperature, Kawate et al. [88] reported oxidation formed by Al_2_O_3_ in similar Cr_1-x_Al_x_N and Ti_1-x_Al_x_N coating systems from ~900 °C and up to 1100 °C. Exposure temperatures of up to 1000 °C promoted significant coating microstructure modifications as nanostructured multilayers started to dissolve. Similar multilayer structural degradation on similar coatings was also reported in [89,90]. A further possible microstructure explanation of the concurring chemical structure modification in the AlTiCr_x_N_1-x_ coating observed from 800 °C is possibly related to the spinodal decomposition of the multilayered coating structure in TiCr-rich compounds that tend to diffuse into the tool steel substrate (according to [91]). To support these arguments, FEG-SEM–EDS analyses were performed across the AlTiCr_x_N_1-x_ coating after exposure to the minimum temperature necessary to form oxide areas (800 °C). A representative such analysis is reported in Figure 8. Thus, this results in a significant presence of Cr at the boundary between the coating and substrate (yellow line in the EDS line scan of Figure 8).

**Figure 9:**
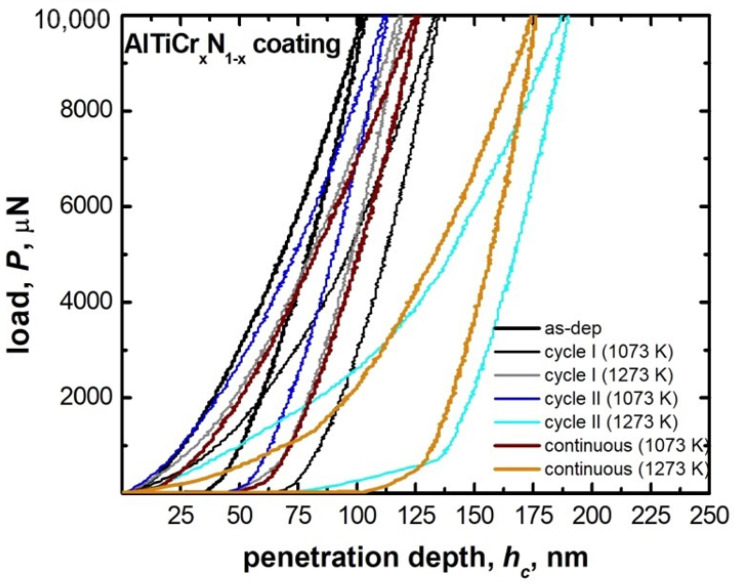
Nanoindentation load-displacement curves of AlTiCr_x_N_1-x_ coating after cycles I, II (see Figure 6), and continuous exposition to 800 and 1000 °C.

Apart from the observed coating surface degradation occurring from 800 °C cycling or long-term (24 h) continuous exposure, a relevant technological aspect refers to the mechanical behavior and response to the same test temperatures. This was inferred by nanoindentation measurements on the AlTiCr_x_N_1-x_ coating, to follow the hardness, *H*, and local elastic modulus (reduced elastic modulus, *E_r_*) at the different test temperatures. Figure 9 reports representative load-displacement nanoindentation curves for cycles I, II and continuous baking (24 h) for two maximum test temperatures (800 and 1000 °C). Penetration depth decreased drastically only in two conditions: namely, cycle II and continuous baking both at the maximum temperature of 1000 °C.

**Figure 10:**
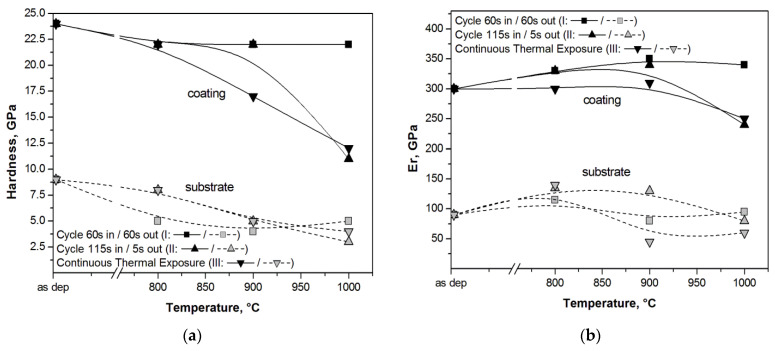
Hardness, *H*, (**a**) and *E_r_* (**b**) of AlTiCr_x_N_1-x_ coating after thermal cycles I, II (see Figure 2), and continuous exposition for 24 h to the upper temperature range (1000 °C). The corresponding behavior of the substrate tool steel is also reported for comparison.

The resulting hardness, *H*, and reduced elastic modulus, *E_r_*, for the different maximum exposition temperatures are plotted in Figure 10.

Initial (as-deposited) hardness of the AlTiCr_x_N_1-x_ coating is almost threefold that of the tool steel. This tends to reduce to almost half at 1000 °C by the most demanding cycle II and by continuously exposing (24 h of duration) the coating to the test temperature. Noteworthily, hardness did not reduce significantly by applying cycle I to the coating, as it remained unchanged at a value of 22.5 GPa (that is only 6% lower than the as-deposited value) for all the temperature ranges 800–1000 °C. Correspondingly, the tool steel hardness reduced by some 50% regardless of the testing conditions followed (i.e., cycled or continuous exposition).

Under the same experimental conditions, the reduced elastic modulus, *E_r_*, of both the coating and the substrate did not change significantly. This revealed that cycle I demanded experimental conditions for the AlTiCr_x_N_1-x_ coating as its elastic modulus did not reduce with maximum cycling test temperatures. As already reported in the case of the hardness comparison between the coating and tool steel substrate, *E_r_* of the coating was threefold that of the substrate, regardless of the experimental conditions.

**Figure 11:**
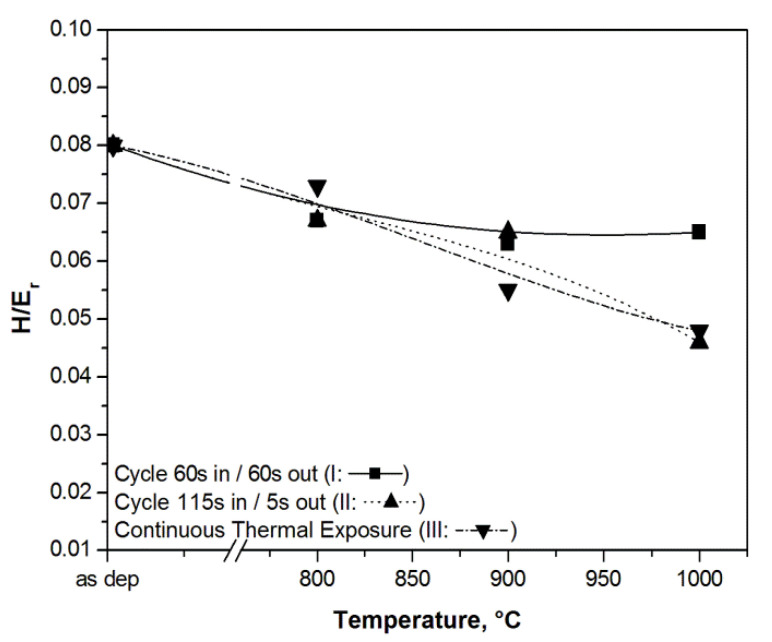
H/Er vs. temperature for cycles I, II, and continuous exposure.

To better assess the mechanical property degradation induced by the maximum exposure temperature to the AlTiCr_x_N_1-x_ coating on tool steel, a technologically meaningful approach is given by the *H/E_r_* ratio. This quantity, indeed, provides an estimation of the coating’s ability to be deformed by pressure without damage [92,93]. The *H/E_r_* quantity refers to the ratio between the plastic work and the total work in an indentation contact, also referred as to the plasticity index (*PI*) and expressed as [25] *PI* = 1 − *η*⋅(*H*/*Er*), where *η* = 6.4. It is generally agreed that the *PI* represents a valid tool to assess the degree of coating degradation. In this regard, the *H/E_r_* trend with testing temperature can give an estimation of the resulting coating degradation. Figure 11 shows plots of the *H/E_r_* ratio against thermal exposition by cycles I, II, and continuously, up to 1000 °C.

As expected, the mechanical degradation described by the PI was more evident by applying the more demanding cycle II and by continuously exposing the coating to the maximum test temperature for 24 h. In these two latter cases, H/Er, and conversely the PI, linearly downgraded to some 40% from 800 to 1000 °C. As per cycle I, the coating downgrading with test temperature was quite low, and it resulted in approximately 10% at its worst for the temperature range 800–1000 °C.

Hence, while cycle II and continuous exposition exhibited similar behavior, in the case of cycle I, the coating ability to plastically deform without damage was retained up to 1000 °C.

This important experimental result showed a much better performance of the present AlTiCr_x_N_1-x_ (with x varying from 0.3 to 0.7) compared to other protective coatings, such ternary AlTiN, AlCrN, TiAlN systems, other similar quaternary AlTiCrN coatings, and even more complex AlTiCr5SiYN systems that can be applied to tool steels [92]. These results can surely be considered meaningful from an industrial and applicative viewpoint. The present AlTiCr_x_N_1-x_ (with x varying from 0.3 to 0.7) can surely be considered a valid alternative to CRE-bearing high-speed tool steels as the surface mechanical properties were quite similar to those of most of the conventional HSS tool steels. In addition, these high mechanical properties were shown to be maintained up to 800 °C without surface oxidation phenomena. Nevertheless, good microstructure stability and mechanical properties were maintained also at working temperatures above 800 °C and by thermal cycling working conditions. In fact, the AlTiCr_x_N_1-x_ coating showed hardness and elastic modulus quite similar to that of the CRE-containing HSS. In addition, the H/Er ratio and thence the plasticity index PI, i.e., the ability to deform without damage, of the AlTiCr_x_N_1-x_ coating on CRE-free tool steels showed promising properties, both in cycling and in continuous high-temperature working conditions at least up to 800 °C.

## 4. Advanced Cast Irons—Austempered Ductile Iron

In recent years, great interest has been given to the development of cast irons with enhanced properties that could be used for different applications, especially in the auto and heavy machinery industry. The properties of new advanced irons are comparable to those of cast or wrought steels or even of aluminum castings regarding specific strength. The enhancement of the cast iron properties is achieved through different additional heat treatments, surface treatments or by the addition of different particles.

Cast iron production is at the level of 73% (76 million tons) of all castings produced worldwide, vastly surpassing aluminum alloys (15%), steel (10%) and other alloys (2%) [93]. Between the cast irons (gray iron, malleable iron, compacted iron, white iron and ductile iron), the use of the ductile irons stands up due to their excellent mechanical properties and the possibility of achieving the highest strength to ductility ratio. This is possible due to the spherical (nodular) appearance of graphite in ductile iron, which introduces the least stress concentration and thus the least weakening of the metal matrix. Furthermore, the metal matrix of ductile irons contains the smallest number of impurities, which further enhances its properties. Further improvement of the ductile iron properties is achieved by an austempering process, thus producing an austempered ductile iron (ADI).

### 4.1. Austempered Ductile Irons

The ADI material has a unique microstructure of ausferrite, which is a mixture of ausferritic ferrite and carbon enriched retained austenite [94,95,96]. Due to this unique microstructure, the ADI materials have a remarkable combination of high strength, ductility and toughness, together with good wear, fatigue resistance and machinability [97,98,99,100]. Depending on austempering parameters, the ADI material can be produced with a tensile strength from 800 MPa to 1600 MPa and elongations from 10% to 1%, respectively [97]. Consequently, the ADI materials are used increasingly in many wear-resistant and tough engineering components in different sectors including automotive, trucks, construction, earthmoving, agricultural, railway and military sectors [101,102].

**Figure 12:**
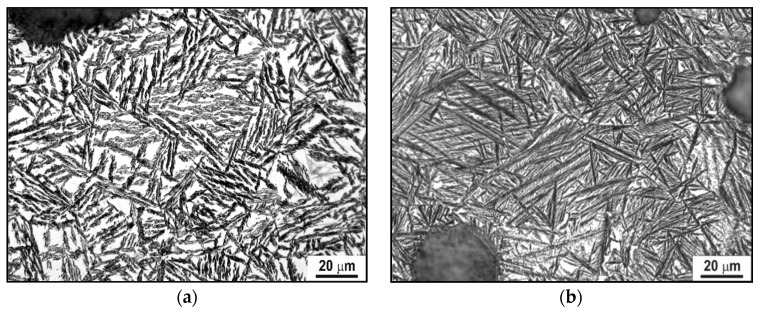
Typical microstructure of ADI (austempered ductile iron) materials: (**a**) plate-like ausferrite morphology (400 °C/2 h); (**b**) acicular morphology (300 °C/2 h).

The production of ADI materials starts from good quality ductile iron with the chemical composition (in wt.%) of 3.6% C (±0.2%), 2.5% Si (±0.2%), 0.35% Mn (±0.05%), ≤0.02% S, ≤0.04% P, (% S × 0.76) + 0.025 ± 0.005, and the addition of other elements for better quenching if necessary (usually for larger sections size): ≤0.60% Mn, ≤0.80% Cu, ≤2% Ni, ≤0.30% Mo [102]. Furthermore, nodularity should be ≥90%, the carbide and inclusion content should be ≤0.5% and the porosity should be ≤1% [101]. The heat treatment process consists of austenitization in the range of 850 to 950 °C for 0.5 to 2 h, usually followed by quenching to austempering at temperatures from 250 to 400 °C, and holding at those temperatures for an appropriate length of time (1 to 2 h). During austempering, the austenite (γ) transforms into a mixture of ausferritic ferrite (α) and carbon enriched retained austenite (γ_HC_), i.e., into the ausferrite. If the casting is held at the austempering temperature too long, then the carbon enriched retained austenite (γ_HC_) starts to decompose into ferrite (α) and carbides [94,95,96]. The formation of carbides in the microstructure makes the material brittle, and hence that reaction should be avoided [96,97]. Subsequently, the optimum mechanical properties of the ADI material can be achieved inside the processing window. The higher austempering temperatures produce an ausferrite microstructure which is more plate-like, with a higher amount of austenite but with less carbon content in austenite (1.2–1.6%C), Figure 12a. On the other hand, lower austempering temperatures produce fine but dense ferrite plates of acicular morphology, Figure 12b. In this case, the carbon content in austenite is higher (1.8–2.2%C), resulting in a more stable austenite. Different ausferrite morphology gives rise to different mechanical properties. The ADI material produced at higher temperatures is more ductile, while the ADI produced at lower temperatures has a higher strength [97,100].

Based on a wide range of properties which could be achieved by austempering of unalloyed ductile iron and producing ADI material, especially considering that the material properties can be enhanced without the application of alloying elements such as Cr, Ni, Mo and Cu in terms of tensile, wear, cavitation erosion and ballistic properties, there are numerous applications in which the unalloyed ADI materials could replace alloyed wrought or cast steels, or carbide (white) cast irons highly alloyed with critical raw materials (CRM), such as Cr, Co, Nb, W, etc.

For that reason, in the following subchapters several possible applications for replacing materials with high amounts of the CRM with the ADI materials will be described.

### 4.2. Wear Properties of the ADI

Due to the excellent combination of properties, the ductile irons and the ADIs are used for a number of applications, some of them relating to equipment exposed to abrasive wear in mining and agricultural industries. Furthermore, the ADI materials could be used as a replacement for high chromium (10 to 20%Cr) white cast irons. In this way, by utilization of unalloyed material, a great impact in reducing the application of CRMs could be achieved.

To assess the wear rate of these materials, two DI (ductile irons), as well as three unalloyed ADI (austempered ductile iron) materials were studied [103]. The microstructure of the DI was fully ferritic or pearlitic (designated as DI-F and DI-P), while the microstructure of the ADI materials produced by austenitization at 900 °C for 2 h, and austempering at 300, 350, 400 °C for 1 h (designated as ADI-300, ADI-350, ADI-400) were fully ausferritic with 16, 24.9 and 31.4% of retained austenite, respectively. The chemical composition of DI-F and subsequent ADI materials produced from DI-F was 3.53% C, 2.53% Si, 0.347% Mn, 0.031% Mg, 0.018% S, 0.015% P, while DI-P had a composition of 3.48% C, 2.19% Si, 0.26% Mn, 1.51% Ni, 1.57% Cu, 0.060% Mg, 0.020% S, 0.012% P. To determine abrasive wear behavior, the pin-on-disc wear tests were performed by SiC grinding paper with grit size P240, P500 and P800, and under 0.5, 1.3 and 2 kg loads.

The increase in the transformation temperature from 300 to 400 °C alters the ausferritic morphology as well as the amount of retained austenite. The microstructure morphology changes from needle-like (acicular) at lower austempering temperatures to a more plate-like (feathery) morphology at higher temperatures. With microstructure, the mechanical properties [103] also changed, Table 3. The ferrite microstructure is soft and ductile, while the fine acicular appearance of the ADI-300 gives high strength and hardness. On the other hand, a large amount of austenite promotes ductility in the ADI-400.

**Figure 13:**
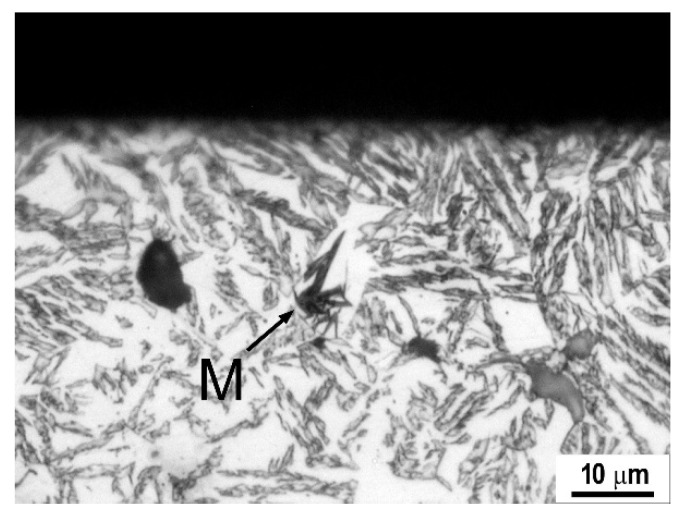
Martensite formation in microstructures of ADI-400 (2 kg, P240 paper, M-martensite).

The microstructure of ADI-300 and ADI-350 did not change after the wear test. On the contrary, martensite formed during wear testing at 2 kg loading and P240 grit paper of the ADI-400. The martensite was formed as a result of local pressure induced by the coarsest grit abrasive particles and maximal loading through stress-assisted phase transformation (SATRAM) (see Figure 13).

The highest wear rate [103] was obtained with the ferrite ductile iron (DI-F), while the lowest was for the hardest ADI-300, Table 4. However, ADI-400, in the case when martensite forms, exhibits better wear resistance. It was found that the wear resistance primarily depends on materials microstructure, corresponding hardness and transformation during wear. In the case of ADI materials, the SATRAM phenomenon plays a major role in wear behavior. However, the SATRAM phenomenon occurs only if appropriate conditions are fulfilled: namely, the presence of metastable, low carbon-enriched retained austenite and sufficient local pressure on the metal matrix.

Finally, to conclude, the microstructure transformation of ADI material gives rise to the possibilities of replacing Cr carbide irons in some applications.

### 4.3. Cavitation Properties of the ADI

Cavitation is characterized by the generation and the collapse of vapour structure in liquid, thus creating shock waves and microjet impacts which finally damage a material. In this kind of extreme working conditions, some highly alloyed (Cr, Ni, Mo, V, Co) steels are usually used; for that reason, finding a suitable alternative is of great importance. As one of the possibilities, an ADI (austempered ductile iron) could be used due to a wide range of mechanical properties (high strength, ductility, toughness, good wear and fatigue resistance) which could be produced by appropriate heat treatment.

Cavitation behavior in water was detailed in a study on the unalloyed ADI material (3.53% C, 2.53% Si, 0.38% Mn, 0.031% Mg, 0.015% S) obtained by 1 h austempering at 300 or 400 °C (ADI-300 and ADI-400) after austenitization at 900 °C for 2 h [104].

The microstructure of ADI materials was fully ausferritic, consisting of a mixture of ausferritic ferrite needles and high carbon enriched retained austenite at 300 °C, and plate-like (feathery) morphology at 400 °C. The amount of retained austenite was 16% and 31.4%, and hardness was 460 and 296 HV30, respectively.

It was found that cavitation damage for both ADI materials was initiated at graphite nodules as well as at pit rims formed by nodule separation. Crack development has a significant influence on cavitation erosion where crack development in ADI austempered at 400 °C occurred at a later stage compared to ADI austempered at 300 °C.

The cavitation rate [104] for ADI-300 was 3.2, 7.5, 11.5, 18.6 and 25.1 mg for an exposure time of 0.5, 1, 2, 3 and 4 h, respectively. The ADI-400 has a cavitation rate of 3.3, 4.3, 8.7, 15.6 and 22.2 mg for the same exposure time period. The slope for unalloyed ADI-300 corresponds to the cavitation rate of 0.1046 mg/min, while the slope of the ADI-400 is 0.0925 mg/min, so the harder ADI-300 material is less resistive to cavitation damage, while the softer ADI-400 material has a higher resistance.

Similar to the previous case, during stress impact of cavitation bubbles, in ADI austempered at 400 °C a low-carbon, retained, metastable reacted austenite transforms through the SATRAM effect into martensite, hence promoting cavitation resistance.

However, it should be pointed out that ADI materials experience additional embrittlement in contact with water [105] and other liquids containing even the smallest amount of water [106]. This is more pronounced for the ADI materials austempered at lower temperatures and it affects elongation the most, while 0.2% yield strength is mostly unaffected. Thus, in the condition of high stress and water contact, the ADI could fracture in the brittle mode.

Finally, to summarize, the microstructure transformation of ADI material gives rise to the possibilities of replacing alloyed steels in some cavitation sensitive applications.

### 4.4. Ballistic Properties of the ADI

The extensively used material for ballistic protection of armored vehicles is armor steel, which contains considerable amounts of critical raw materials (CRMs), such as chromium (up to 2%) and molybdenum (up to 1%). By replacing the steel with unalloyed heat-treated ductile iron (austempered ductile iron—ADI material) as additional perforated armor, considerable savings could be achieved regarding cost, weight and reduction in the use of CRMs.

The ADI materials have the strength and hardness comparable to some types of armor steels, while their ductility is somewhat lower. Furthermore, their density is also lower, which, along with a lower cost, makes them an attractive engineering material. Therefore, ADI materials can be regarded as an effective replacement for steel, particularly when a lower ductility can be tolerated.

The study [107] was to replace a 6 mm alloyed steel perforated plate with an unalloyed ADI material perforated plate in order to increase the ballistic protection of the basic steel plate. 

Two types of unalloyed ADI materials were prepared, austenitized at 900 °C for 2 h and austempered at 275 and 400 °C for 1 h (designated ADI-275 and ADI-400). The first was harder with a higher strength, while the second had a higher ductility, Table 5. The as-cast material was unalloyed ductile iron with a chemical composition as follows: 3.51% C, 2.21% Si, 0.38% Mn, 0.19% Cu, 0.031% Mg, 0.035% P, 0.014% S. 

The microstructure of the ADI materials (ADI-275 and ADI-400) was fully ausferritic consisting of a mixture of ausferritic ferrite and retained austenite. The amount of austenite was 9.8 and 26%, respectively. Furthermore, due to the austempering temperature, two different morphologies of microstructures were obtained: the needle-like acicular type at the 275 °C austempering temperature and the more plate-like, feathery type at the 400 °C.

Ballistic testing specimens were assembled from heat-treated sections which were firmly mounted on two steel frames at 400 mm from the basic 13 mm RHA (rolled homogenous armor) plate, both set at 0° from vertical. This arrangement was intended to improve the basic RHA protection level from 7.92 × 57 mm SmK (Spitzgeschoss mit Kern) hardened steel core to 12.7 × 99 mm M8 armor-piercing incendiary (API) ammunition. The sections had thicknesses of 7 and 9 mm, and the drilled holes with a diameter of 11 mm and ligament length of 3.5 mm (center to center distance of 14.5 mm).

The appearance of the ballistic damage to the perforated plates was different. In the case of the ADI-275, a larger area had a fracture of approximately five to six interconnected holes, but with small deformation. Whereas the ADI-400 fractured with pronounced inward deformation, but on a smaller interconnected area of approximately three to four holes. An intensive plastic deformation during the impact of ADI-400 caused a SITRAM (strain-induced phase transformation of retained austenite into martensite) effect to occur in the material, causing a partial brittle fracture and a lower ballistic performance.

It was found that only ADI material austempered at 275 °C, 7 and 9 mm thick, offers full ballistic protection of the basic 13 mm armor steel against 12.7 mm armor-piercing incendiary ammunition, which refers to five projectiles stopped by the basic plate. Both the 7 mm and 9 mm perforated plates austempered at a lower temperature of 275 °C producing higher hardness and lower ductility were effective in fracturing the penetrating core, thereby significantly decreasing the chances of penetrating the basic plate.

Thus, the perforated plates made of the ADI-275 can be regarded as a more economical replacement with similar mass effectiveness as perforated plates made of alloyed steel of a 6 mm thickness.

Further savings could be achieved by utilizing an austempered compacted cast iron (ACGI), which does not possess spheroidal graphite but contains graphite of a short and broad lamellar type due to a lower amount of spheroidisers/nodularisers (cerium and magnesium) [108]. The ACGI compared to ADI material has lower production costs and a lower amount of magnesium, thus reducing the application of CRM. However, a larger damaged area with a greater number of interconnecting holes is likely to provide the ACGI perforated plate poor multi-hit resistance compared to steel or the ADI perforated plates [109], and further study is needed in order to optimize its application.

### 4.5. Summary of the ADI Properties

As it is demonstrated in the aforementioned sections (Section 4.1, Section 4.2, Section 4.3 and Section 4.4), the application of unalloyed austempered ductile irons, such as advanced cast iron, has numerous possibilities for different applications in order to replace or reduce the use of CRMs by substituting alloyed steels.

The most critical parameter in optimizing ADI microstructure, and thus its application, is the stability of retained austenite and transformation of the austenite to martensite by the SITRAM/SATRAM effect (strain-induced or stress-assisted phase transformation of retained austenite into martensite). The formation of martensite has a positive effect on wear and cavitation behavior, while it hinders ballistic applications.

## 5. Iron-Based Materials as Solutions for CRM Substitution in Other Tool Materials

Cemented carbide, i.e., the material composed of tungsten (or another carbide) connected by cobalt binder is one of the most widely applied tool materials. Due to an excellent combination of wear resistance and fracture toughness, it finds its use in the machining of non-ferrous metals, drilling of concrete and many other applications. Since both tungsten and cobalt are listed as CRMs, the substitution of this material with the simultaneous keeping of the properties is a dream of many scientists. In general, there are three possible approaches:

- Substitution of tungsten carbide;
- Substitution of cobalt binder;
- Design of a whole-new concept of CRM-free material.

The first two approaches are described in detail in [12]. For the abrasive wear conditions, the solution could be the use of intermetallics since some of them are very hard. In recent years, the Fe–Al–Si material containing a high amount of silicon (Fe-20Al-20Si in wt.%) was tested for wear resistance. It has been found that this material possesses a similar or slightly better wear resistance than AISI D2 tool steel [109]. It still does not reach the wear performance of cemented carbide and has a lower fracture toughness at room temperature, but the material is characterized by exceptional thermal stability and anomalous temperature dependence of yield strength and ultimate tensile strength; i.e., the mechanical properties increase with the temperature in some intervals (400–600 °C) [109].

**Figure 14:**
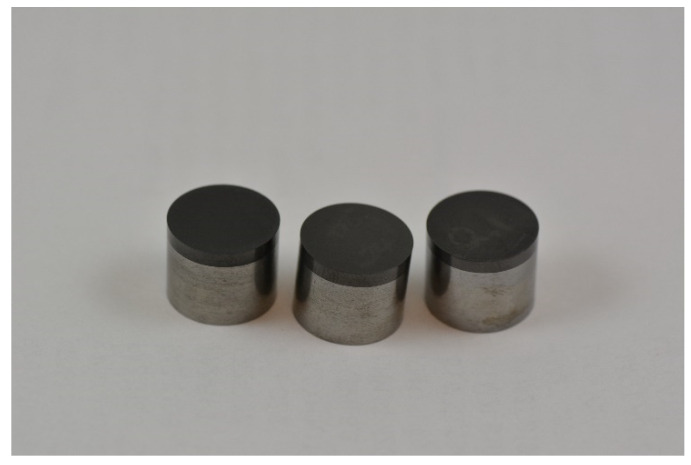
Inserts for drill tools drill tools, which consist of a polycrystalline diamond layer (black colour) on a cemented carbide substrate, inserts diameter is 12.5 mm.

Drilling and processing of natural stone and concrete requires high-tech tools, which can be divided into two groups. The first group comprises polycrystalline diamond materials mainly used as inserts for drill tools. Polycrystalline diamond compact bits are one of the most widely used oil and gas drilling tools, Figure 14. 

The second group contains diamond saws and grinding wheels, which are widely used in construction for cutting, grinding, drilling holes in masonry and concrete, cutting of asphalt, curbs, sidewalks, railway sleepers, concrete, stone and the demolition of buildings, etc. 

In both groups of materials, cobalt is used as the basic binder material. Polycrystalline diamonds (PCD) have been explored since the 1980s for replacing the cobalt binder with another metallic or ceramic bonding phase [110,111,112].

It is much more difficult to substitute cobalt in metallic-diamond materials for saws and grinding wheels. Building stone and construction materials are highly abrasive and therefore require tools with the best cutting properties and high abrasion resistance. The production of tools for the processing of building stone includes:

- Tools for extracting large rock blocks in quarries;
- Tools for cutting rock blocks;
- Tools for cutting and processing cladding and tiles;
- Stone grinding and polishing tools.

**Figure 15:**
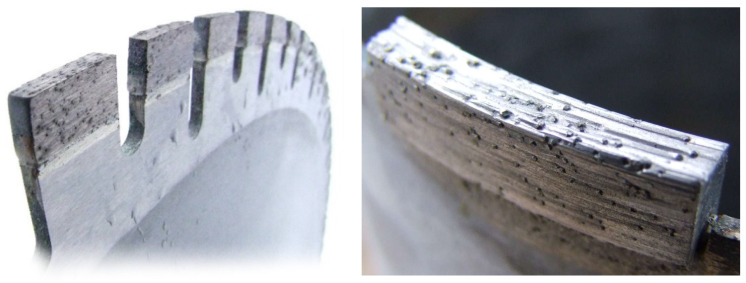
Examples of sections of the newly made saws. Diamond segments: the segment dimension is dependent on the saw diameter. For the presented Ø450 mm saw, the segments dimension is 40 × 4.2 × 10 mm.

**Figure 16:**
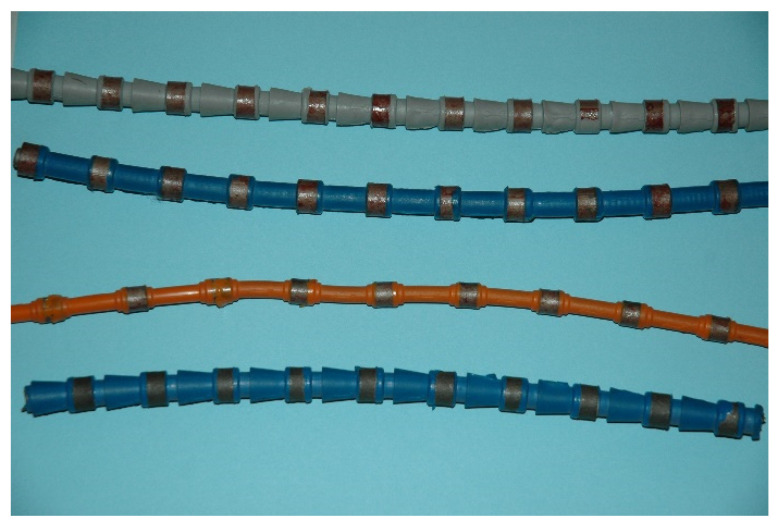
Examples of polycrystalline diamond wires, in the figure there are various diamond wire saws which are characterized by the number of wire beads (40–28 pcs/m) and the diameter of beads (11.5–6.3 mm).

Metallic-diamond materials, constituting the working elements of the saws used in the processing of natural stones, are most often evenly distributed on a rope, tape or disc, Figure 15. These are wire saws, gang saws or circular saws; see Figure 16. 

Wire saws have come into common use for cutting rock blocks from a deposit [113]. Gang saws are used to cut large blocks of rock to obtain plates of equal thickness [114]. The working principle of such a saw is that it makes a reciprocating movement with its simultaneous “falling”. These two types of saws are used in quarries. On the other hand, circular saws are the most commonly used tools for processing natural stones [115]. Metallic-diamond materials, which are the working elements of tools used for cutting and processing stones, should be durable and ensure operation with the lowest possible energy consumption [116,117].

In tool operation, the matrix must ensure sufficient retention of the diamond particles, i.e., have high retention properties and wear at a rate comparable to the wear rate of diamond particles [118]. This guarantees the appropriate speed of revealing new diamond particles with sharp cutting edges and thus maintaining the continuity of the cut [117,119,120]. The matrix must maintain the diamond particles in such a way as to prevent them from falling out, pressing in or turning during cutting. In practice, the selection of the matrix depends on many factors: the type of diamond, the size of the diamond particles, the strength of the diamond particles, cutting conditions and the properties of the processed material [121]. Due to the very different working conditions of metallic diamond tools, a wide variety of materials are used as the matrix. Matrices based on cobalt, copper, bronze, tungsten, tungsten carbide or their mixtures are in wide application. If the matrix is too soft with low abrasion resistance, it can wear very quickly, leading to excessive diamond dropout. When the matrix shows high abrasion resistance, the tool surface may be polished, and the cutting process may be interrupted [118].

Diamonds are impregnated in the metal matrix where the connection between the matrix and the diamonds must be strong enough. Cobalt is the most commonly used matrix for metal-diamond tools intended for cutting hard natural stones. It ensures tool wear at a speed comparable to that of diamond particles, as well as very good retention properties associated with favourable strength properties [122]. Cobalt and W–Co alloys are used for high hardness materials such as granite. Co, Co–bronze, iron–Co and Fe–bronze binders are used in cutting materials of moderate hardness, such as marbles. Research on limiting the use of cobalt in diamond tools began very early at the beginning of the 21st century [123,124,125]. The research was carried out by large companies producing metal bond matrices and tools for a reason similar to today’s causes. Now, the quest for new matrix materials is motivated by the fact that cobalt is a critical raw material. Twenty years ago, the fluctuating and still rising cobalt prices on world markets were pointed out as the cause. Scientific research on the substitution of cobalt by a different type of binder was also carried out due to its harmfulness to human health [126,127]. Most of the world’s producers of diamond blades, core bits and other tools for the processing of natural stone and construction materials use pre-alloyed powders. The sintering technique for this type of material has changed, and the hot pressing (HP) method has become dominant. Using this method, the sintering time was significantly reduced compared to free sintering. Therefore, the binder powders must be pre alloyed. Products from well-known companies include NEXT^®^100 (50 wt.% Cu-25 wt.% Fe-25 wt.% Co), Cobalite^®^ HDR (Fe-7 wt.% Cu-27 wt.% Co) and DIABASE^®^ (45–65 wt.%)Fe-40 wt.% Cu-(20–40) wt.% Co, KEEN^®^ [128,129,130,131]. Most of the compositions of these commercial matrices are based on the Fe–Cu–Co mixture, with different compositions of elements. These powders are mixed with diamonds, then diamond saw segments are cold pressed and sintered usually by hot pressing (HP) or free sintering techniques. The bond material is selected depending on the material that needs to be cut. A phase diagram was developed for the Fe–Cu–Co system at a temperature of 850 °C, which corresponds to the manufacturing temperature of sintering diamond tools by the HP method [125]. Microstructural studies confirmed that for this sintered alloy, a FeCo solid solution is embedded into a Cu matrix. The Cu solubility is limited to about 2% at 800 °C [132,133]. A fairly good agreement has been achieved between the developed thermodynamic description and experimental data from XRD measurements and microstructure analysis found in the literature. A new correction of the ternary Co–Cu–Fe systems has been performed using the CALPHAD method [134].

The Fe–Co solid solution is essential for commercial matrices, which is responsible for good tool properties because it strengthens the alloy [133]. These matrices contain most commonly about 20% cobalt, which is still a high content, and therefore the research on total cobalt substitution continues. The most suitable candidates for cobalt substitution are ferrous alloys with nickel or manganese, copper, tin and carbon. New solutions of binders are related to the Fe–Cu phase equilibrium system. The iron–copper system does not form amorphous phases or metastable solid intermetallic compounds and has negligible mutual solid dissolution phases in the temperature range corresponding to tool making [135,136]. However, the properties of alloys selected from the Fe–Cu system are not as good as cobalt-containing binders [137]. Diamond tools were prepared by mixing 85 wt.% of Fe–Cu alloys (60 wt.% elemental Fe and 40 wt.% elemental Cu) with 15 wt.% Fe-P. Tools with the Fe–P alloy as reinforcement, have wear behaviour similar to tools with a cobalt bond [138]. Up to 2 wt.% Si was added to the cobalt-containing binders with good results in improving the adhesion of the bonding phase to diamond [139]. To increase the abrasion resistance of the binder, 0.5–2 wt.% WC was introduced [140]. These two concepts were combined, and SiC was introduced into the Fe-Cu binder. Despite good results, it was found that this binder cannot compete with the binder with a high content of cobalt. The phase composition and microstructure of the Fe–Cu binders are influenced by the content of copper and these bonds are very sensitive to the heat treatment [141]. By creating solid solutions and carbides, the iron forms a strong bond with the diamond [142]. The level of diamond degradation caused by iron is higher than that caused by cobalt and nickel. Copper is carbon inert and no chemical reactions with diamond are visible [143]. The studies related to the modification of the properties of the Fe–Cu binder by introducing metal additives continue. However, due to the limitation resulting from the diamond graphitization process, the most commonly used additives are low-melting metals. Diamond is an allotropic form of the element carbon with a cubic structure, which is thermodynamically stable at pressures above 1.6 GPa at room temperature and metastable at atmospheric pressure. The thermal resistance of natural diamond is higher than that of synthetic diamond crystals. Additives such as Co, Ni and Fe are catalysts for the conversion of graphite to diamond, and therefore their presence accelerates the reverse conversion. Generally, the temperature of the graphitization process is affected by the type of binder [143,144]. TiC coatings on diamond particle surfaces limit the graphitization process of diamond in the tools, which is accelerated by the Fe (the catalyst of diamond transition into graphite [145]).

At present, materials are more complex: additional elements such as tin and zinc are introduced in small amounts, for example, 48-53 wt.% Fe-40-45 wt.% Cu-1-3 wt.% Sn-2-4 wt.% Zn, and the matrix has a suitable retention mechanism with diamond [146]. The Fe–Cu–Ni binders are another group of iron-based binders. These materials have slightly lower strength and ductility compared to cobalt, but hardness and offset yield strength are sufficiently high [147]. Fe–Ni–Cu–Sn–C and Fe–Mn–Cus–Sn–C alloys are potential Co–WC substitutes for the manufacture of sintered diamond-impregnated tools for abrasive applications [148]. The properties of these materials could be modified by changing the milling conditions [149]. Commercial powders from the Fe–Cu–Re system are available; research related to their application as the bond of diamond tools has shown that the properties of these binders are not better than that of cobalt binders and could be used for less demanding applications [150]. A separate group of actions to improve the properties of diamond tools is the strengthening of the Fe–Cu–Ni–Sn binder by introducing particles of hard ceramic materials. For example, this bond with 2 wt.% CrB2 has very good tribological properties [151]. Ball-milled, sintered Fe–Ni–Cu–Sn–C, Fe–Ni–Cu–Sn–C-Al_2_O_3_ and Fe–Ni–Cu–Sn–C–Al_4_C_3_ materials showed fine-grained microstructures and significant strengthening due to a martensitic transformation [152]. Very promising results were obtained for the use of the dispersion strengthened Fe–Ni–Mo matrix by nanoparticles of WC, hBN and nanotubes [153].

Historically, cobalt has been the ultimate bonding agent for polycrystalline diamond tools. Pre-alloyed materials from the Fe–Cu–Ni system are promising as binders in tools intended for stone processing. This matrix, however, must be strengthened by introducing metal additives: most often low-melting metals or particles of hard materials. These matrices exhibit adequate abrasion resistance and diamond retention, comparable to cobalt-containing binders.

## 6. Iron-Based High-Temperature Alloys as CRM Substitutes

After World War II, some European countries were facing severe problems in buying chromium (especially low carbon ferrochrome) and deficit nickel on the free market to cover their needs for stainless steel production and materials for high temperature (HT) use. To overcome this shortage, Russia (formerly the Soviet Union) and the Czech Republic (formerly Czechoslovakia) initiated a strong partnership in the 50 s between national research institutions, universities and local industry to develop low-cost alternatives for heat resistant steel-alloys based on intermetallics cast from accessible and cheap iron, aluminum and carbon. These efforts resulted in the past in the materials such as Thermagal in France, Tchugal in the Soviet Union and Pyroferal in Czechoslovakia. Very important results were achieved in the research between 1950 and 1962, based on the numerous research reports of J. Pluhař, M. Vyklický and H. Tůma—the scientists who initiated and completed the research leading to the development of Pyroferal [154].

Pyroferal offered quite impressive results on high temperature corrosion resistance. It was tested against air atmosphere, vanadium pentoxide, molten glass, carburization, nitration and the atmosphere of natural gas cracking generators. Good results were also described on wear resistance and hardness due to the appearance of hard and brittle aluminum carbide Al_4_C_3_ and perovskite phase Fe_3_AlC. Though Pyroferal was manufactured only by casting, welding was an important procedure not only for producing complicated shapes, but also for repairing the faults in casts. Unfortunately, the practical use of these materials was limited due to various problems: e.g., the stability of the perovskite phase in the air at room temperature (RT), substantial shrinkage during the manufacturing of casts, conditional weldability. For welding, the components have to be heated to 650 °C and held at temperature for at least another 15 min with subsequent slow cooling to RT. These problems could not be overcome by the state-of-the-art in material science at that time. Typical products were puddle blades of pyrite calcining furnaces, parts of clotting furnaces, carburizing charger grids, steam furnace bars, parts of glass furnaces, rails of tunnel furnaces and parts of fluid combustion chambers, stator blades of internal combustion turbines, a carburization chamber and an experimental work holder for the cog-wheel of a differential gear from the end of the 90 s. In the 1960s, access to chromium was not a problem any longer, and the dust of history covered the know-how on (pre-)industrial use of FeAl. In the half nineties of the last century, we can see the rebirth of interest in this type of material [154]. However, after a short time, Pyroferal-type material was replaced by materials based on FeAl and Fe_3_Al with a small amount of additives. It was caused by the state of science and science possibilities, which allowed a shift to the next generation of Fe–Al intermetallic materials.

As the additives to Fe–Al alloys, the common alloying elements of steel (chromium, nickel, molybdenum) are considered, as well as zirconium or niobium [155]. However, some of these elements are listed as CRMs (niobium, zirconium) or have been listed recently and are still considered as strategic (chromium). The new approach is the addition of silicon, forming the new generation of Fe–Al–Si alloys. Silicon strongly improves the oxidation resistance of the Fe–Al based alloy. A very interesting fact is that the oxidation resistance does not depend significantly on the silicon content when the level of behavio. 5 wt.% is exceeded [156]. The mechanism, how the silicon improves the oxidation behavior, can be summed up as the following: The Fe–Al based alloy oxidizes, forming the Al_2_O_3_ layer with a minor amount of Fe_2_O_3_. As the material beneath the oxide layer becomes depleted by aluminum, silicon concentrates there and a silicide layer is formed. This layer acts as the secondary protection against the penetration of both oxygen and nitrogen [156,157]. These new alloys can be processed by innovative powder metallurgy processes (mechanical alloying and spark plasma sintering to reach higher mechanical properties [158]). The compressive strength of the alloys processed by these methods exceeds 1500 MPa and even increases with increasing temperature up to 600 MPa. Besides, the alloys achieve very high wear resistance, comparable with heat-treated tool steel AISI D2 [109]. However, low room temperature ductility remains a limitation to the use of these alloys, as well as many other intermetallics.

Besides room temperature ductility, a possible limitation is also the fact that silicon, needed for the production of these alloys, is listed as a CRM. However, it could be easily and cheaply supplied from scrap. In addition, it solves a big recycling problem. When recycling the Al–Si alloy castings (common casts containing pressed-in iron components, e.g., covers containing bearings, bushings, but the best example is the car engine block), the content of iron could become very high and hence the alloy is unsuitable for secondary production, which causes its low price (approx. 10 times lower than aluminum). Iron cannot easily be removed from the alloy and therefore the secondary alloy is usually just diluted by pure primary aluminum. As an alternative, the physicochemical processes have been developed to reduce the iron content in the secondary raw material, even though they are not highly efficient. One such process deals with annealing of the aluminum alloy in the semisolid state (at a temperature between solidus and liquidus temperature) for a longer time, enabling the solid Fe–Al–Si intermetallics to settle down [159]. The Fe–Al–Si concentrate from this process, or even the high-iron Al–Si based scrap itself, could be the source of silicon for the production of new grades of Fe–Al–Si alloys. 

## 7. Conclusions

The possibilities and strategies for partial or total substitution of critical raw materials in iron-based alloys were comprehensively assessed and reviewed. It can be seen that most of the elements are not so easily replaceable due to their specific functions. For example, chromium which was recently listed as a critical raw material and still has to be considered strategic, cannot be fully substituted in stainless steel. However, it can be suppressed by additional mixing of aluminum and silicon. However, silicon is yet another CRM. A similar situation exists in the case of the substitution of tungsten and vanadium in tool steel. These carbide-forming elements could potentially be substituted by niobium or titanium, but both of these elements are also critically listed at present. An improved understanding of the complex transformation processes involved in the processing would allow minimizing the use of and recycling rare earth elements. Coatings were identified as another useful way to upgrade lower grade tools and low-Cr steel to the surface parameters of high-speed and corrosion resistant steel, thus reducing the consumption of CRMs. In other tool materials, iron-based alloys could be the substitutes for the cobalt binder in stone-cutting diamond tools. New grades of iron-based intermetallics are candidates for high-temperature alloys. Austempered ductile cast iron was presented as the material with interesting properties, which could be a substitute for steel and other CRM-containing alloys.

## Figures and Tables

**Table 1:** 2020 EU Critical Raw Materials list (* N 2020—CRMs in 2020, non-CRMs in 2017, ** HREEs—heavy rare earth elements, *** LREEs—light rare earth elements, **** PGMs—platinum group metals).

Materials and Elements			
Antimony (Sb)	Fluorspar	Magnesium (Mg)	Silicon Metal (Si)
Baryte	Gallium (Ga)	Natural Graphite	Tantalum (Ta)
Bauxite—N 2020 *	Germanium (Ge)	Natural Rubber	Titanium (Ti)—N 2020
Beryllium (Be)	Hafnium (Hf)	Niobium (Nb)	Vanadium (V)
Bismuth (Bi)	HREEs **	PGMs ****	Tungsten (W)
Borates	Indium (In)	Phosphate rock	Strontium (Sr)—N 2020
Cobalt (Co)	Lithium (Li)—N 2020	Phosphorus (P)	
Coking Coal	LREEs ***	Scandium (Sc)	

**Table 2:** Chemical composition of the powder metallurgically processed high-speed steel (Böhler grade S290 PM).

Component	C	Si	Mn	Cr	Mo	V	W	Co	Fe
Mass fraction in %	2.0	0.44	0.27	3.7	2.4	5.1	14.1	11.0	bal.

**Table 3:** Mechanical properties of the DI and ADI materials used for wear test.

Material	0.2% Yield Strength [MPa]	Tensile Strength [MPa]	Elongation [%]	Un-Notched Impact Energy [J]	Hardness HV10
DI-F	314	433	27.8	144	161
DI-P	677	880	3.2	20.5	296
ADI-300	1395	1513	3.8	68	470
ADI-350	1071	1221	8.2	108	365
ADI-400	757	1042	14.2	140	306

**Table 4:** Dependance of the wear rates [mg/min] on applied load [kg] and paper grit (P).

Load	Paper Grit	DI-F	DI-P	ADI-400	ADI-350	ADI-300
0.5 kg	P240	34.8	23.8	15.6	13.9	12.6
	P500	21.8	15.5	7.7	7.2	5.3
	P800	6.9	5	4	3.4	2.9
1.3 kg	P240	78.3	60	35.1 *	35.6	34.7
	P500	22.7	17.3	14.3	12.7	10.1
	P800	17.2	13.8	8.7	7.1	5.5
2 kg	P240	108.6	81	43.4 *	52	41.4
	P500	57.1	36.8	18.3	16.9	14.7
	P800	22	20	15.8	13.8	10.3

* Lower wear rates due to formation of martensite.

**Table 5:** Mechanical properties of the ADI materials used for ballistic test.

Material	0.2% Yield Strength [MPa]	Tensile Strength [MPa]	Elongation [%]	Un-Notched Impact Energy [J]	Hardness HV10
ADI-275	-	1472	1	23	498
ADI-400	679	914	8	44	300

## Data Availability

This is the review paper with minor amount of unpublished results of the authors. These data are stored by the authors, not available publically.

## References

[1] European Commission (2020). A New Industrial Strategy for Europe.

[2] European Commission (2011). Tackling the Challenges in Commodity Markets and On Raw Materials.

[3] European Commission (2008). Policy and Strategy for Raw Materials.

[4] European Commission and Directorate_General_Joint_Research_Centre (2017). Methodology for Establishing the EU List of Critical Raw Materials. Guidelines.

[5] (2020). Study on the EU’s list of Critical Raw Materials—Final Report.

[6] Pippel E., Woltersdorf J., Pöckl G., Lichtenegger G. (1999). Microstructure and Nanochemistry of Carbide Precipitates in High-Speed Steel S 6-5-2-5. Mater. Charact..

[7] Dobrzański L.A., Kasprzak W. (2001). The influence of 5% cobalt addition on structure and working properties of the 9-2-2-5, 11-2-2-5 and 11-0-2-5 high-speed steels. J. Mater. Process. Technol..

[8] (2020). Study on the EU’s list of Critical Raw Materials Non-Critical Raw Materials Factsheets.

[9] Working Group on Defining Critical Raw Materials for EU (2014). Report on Critical Raw Materials for EU. http://mima.geus.dk/report-on-critical-raw-materials_en.pdf.

[10] (2017). Study on the Review of the List of Critical Raw Materials—Criticality Assessments. https://op.europa.eu/en/publication-detail/-/publication/08fdab5f-9766-11e7-b92d-01aa75ed71a1.

[11] (2017). Study on the Review of the List of Critical Raw Materials—Non-Critical Raw Materials Factsheets.

[12] Grilli M.L., Bellezze T., Gamsjäger E., Rinaldi A., Novak P., Balos S., Piticescu R.R., Ruello M.L. (2017). Solutions for Critical Raw Materials under Extreme Conditions: A Review. Metals.

[13] Han J., Li Y., Jiang Z., Yang Y., Wang X., Wang L., Li K. (2013). Summary of the Function of Sn in Iron and Steel. Adv. Mat. Res..

[14] Davis J.R. (1994). Stainless Steel—ASM Specialty Handbook.

[15] Di Caprio G. (2003). Gli Acciai Inossidabili.

[16] Van Rooyen G.T. The Potential of Chromium as an Alloying Element. Proceedings of the 1st International Chromium Steel and Alloys Congress.

[17] (1987). Metals Handobook, Corrosion.

[18] Cunat P.J. (2004). Alloying Elements in Stainless Steel and Other Chromium-Containing Alloys.

[19] Floreen S. (1982). An Examination of Chromium Substitution in Stainless Steels. Metall. Trans. A.

[20] Bittence J.C. (1989). Can There Be “Stainless” Without Chromium?. Mater. Eng..

[21] (1985). Substitution Alternatives for Strategic Materials. Strategic Materials: Technologies to Reduce US Import Vulnerability.

[22] Glenn M.L., Larson D.E. (1984). Reduced-Chromium Stainless Steel Substitutes Containing Silicon and Aluminum.

[23] Bullard S.J., Larson D.E., Dunning J.S. (1992). Oxidation and Corrosion Resistance of Two Fe-8Cr-16Ni-Si-Cu Alloys. Corrosion.

[24] Dunning J.S., Alman D.E., Rawers J.C. (2002). Influence of Silicon and Aluminum Additions on the Oxidation Resistance of a Lean-Chromium Stainless Steel. Oxid. Met..

[25] Engkvist J., Bexell U., Grehk M., Olsson M. (2009). High temperature oxidation of FeCrAl-alloys-Influence of Al-concentration on oxide layer characteristics. Mater. Corros..

[26] Wolff I.M., Iorio L.E., Rumpf T., Scheers P.V.T., Potgieter J.H. (1998). Oxidation and corrosion behaviour of Fe-Cr and Fe-Cr-Al alloys with minor alloying additions. Mater. Sci. Eng. A.

[27] Jönsson B., Lu Q., Chandrasekaran D., Berglund R., Rave F. (2013). Oxidation and Creep Limited Lifetime of Kanthal APMT®, a Dispersion Strengthened FeCrAlMo Alloy Designed for Strength and Oxidation Resistance at High Temperatures. Oxid. Met..

[28] Pothen F., Goeschl T., Löschel A., Jaha V. (2013). Strategic Trade Policy and Critical Raw Materials in Stainless Steel Production.

[29] Cavallini M., Felli F., Fratesi R., Veniali F. (1982). High temperature air oxidation behaviour of “poor man” high manganese-aluminum steels. Mater. Corros..

[30] Casteletti L.C., Neto A.L., Totten G.E., Heck S.C., Fernandes F.A.P. (2010). Use of Fe-31Mn-7.5Al-1.3Si-0.9C Alloy for Fabrication of Resistive Elements. J. ASTM Int..

[31] Bellezze T., Giuliani G., Roventi G., Fratesi R., Andreatta F., Fedrizzi L. (2016). Corrosion behaviour of austenitic and duplex stainless steels in an industrial strongly acidic solution. Mater. Corros..

[32] Chen W.Y.C., Stephens J.R. (1979). Anodic Polarization Behaviour of Austenitic Stainless Steel Alloys with Lower Chromium Content. Corrosion.

[33] Glenn M.L., Bullard S.J., Larson D.E., Rhoads S.C. (1985). Partial replacements of chromium in stainless steel. J. Mater. Energy Syst..

[34] Hio K., Yamada T., Tsuchida Y., Nakajima K., Hosoi Y. (2002). Effect of Chromium Content on Anodic Polarization Characteristics of Fe-Cr-Al and Fe-Cr-Si Alloys. Corrosion.

[35] Bellezze T., Giuliani G., Roventi G. (2018). Study of stainless steels corrosion in a strong acid mixture. Part 1: Cyclic potentiodynamic polarization curves examined by means of an analytical method. Corros. Sci..

[36] Sheirer L.L., Jarman R.A., Burnstein G.T. (1994). Stainless Steels. Corrosion—Metal/Environment Reactions.

[37] Davis J.R., Davis and Associates (1994). Atmospheric and Aqueous Corrosion. ASM Speciality Handbook—Stainless Steels.

[38] Abdul-Azim A.A., Rahem Ghanem W.A.E., Abou-Shahba R.M. (1994). Corrosion behaviour of low-Cr high·Al stainless steels in 65% boiling HNO3. Steel Res..

[39] Reformatskaya I.I., Rodionova I.G., Podobaev A.N., Ashcheulova I.I., Trofimova E.V. (2006). Silicon as an Alloying Element in Ferrite Stainless Steels Containing 8–13% Cr. Prot. Met..

[40] Hodgkiess T., Chia P.S. (1991). Assessment of lower-alloy stainless steels for use in desalination plant. Desalination.

[41] Basile F., Lorthioir G. (1993). Quantitative analysis, by cathodic reduction, of passive layers on Fe-17Cr alloy and its application to substituted alloys. Brit. Corros. J..

[42] Wan J., Ran Q., Li J., Xu Y., Xiao X., Yu H., Jiang L. (2014). A new resource-saving, low chromium and low nickel duplex stainless steel 15Cr-xAl-2Ni-yMn. Mater. Des..

[43] Cavallini M., Felli F., Fratesi R., Veniali F. (1982). Aqueous solution corrosion behaviour of “poor man” high manganese-aluminum steels. Mater. Corros..

[44] Abuzriba M.B., Musa S.M. (2015). Substitution for chromium and nickel in Austenitic stainless steels. Springer Proceedings in Physics, Proceedings of the 2nd International Multidisciplinary Microscopy and Microanalysis Congress Oludeniz, Turkey, 16–19 October 2014.

[45] Moon J., Ha H.-Y., Kim K.-W., Park S.-J., Lee T.-H., Kim S.-D., Jang J.H., Jo H.-H., Hong H.-U., Lee B.H. (2020). A new class of lightweight, stainless steels with ultra-high strength and large ductility. Sci. Rep..

[46] Tandon V., Patil A.P., Rathod R.C. (2019). Enhanced corrosion resistance of Cr-Mn ASS by low temperature salt bath nitriding technique for the replacement of convectional Cr-Ni ASS. Anti-Corros. Methods Mater..

[47] Li C., Bell T. (2006). Corrosion properties of plasma nitrided AISI 410 martensitic stainless steel in 3.5% NaCl and 1% HCl aqueous solutions. Corros. Sci..

[48] Sakasegawa H., Tanigawa H., Ando M. (2014). Corrosion-resistant coating technique for oxide-dispersion-strengthened ferritic/martensitic steel. J. Nucl. Sci. Technol..

[49] Bobzin K., Zhao L., Öte M., Königstein T. (2019). Development of a FeCrMnBC-based economical wear and corrosion resistant coating. Surf. Coat. Technol..

[50] Kotrba A., Quan T., Wei W., Detweiler Z., Keifer D., Bullard D. (2020). Spatially Optimized Diffusion Alloys: A Novel Multi-Layered Steel Material for Exhaust Applications. SAE Int..

[51] Bellezze T., Roventi G., Quaranta A., Fratesi R. (2008). Improvement of pitting corrosion resistance of AISI 444 stainless steel to make it a possible substitute for AISI 304L and 316L in hot natural waters. Mater. Corros..

[52] Parsons S., Poyntz-Wright O., Kent A., McManus M.C. (2019). Green chemistry for stainless steel corrosion resistance: Life cycle assessment of citric acid versus nitric acid passivation. Mater. Today Sustain..

[53] Balzar D., Ledbetter H. (1995). Accurate Modeling of Size and Strain Broadening in the Rietveld Refinement: The “Double-Voigt” Approach, Advances in X-Ray Analysis 38.

[54] Wiessner M., Gamsjäger E., Van Der Zwaag S., Angerer P. (2017). Effect of reverted austenite on tensile and impact strength in a martensitic stainless steel ? An in-situ X-ray diffraction study. Mater. Sci. Eng. A.

[55] Wießner M., Leisch M., Emminger H., Kulmburg A. (2008). Phase transformation study of a high speed steel powder by high temperature X-ray diffraction. Mater. Charact..

[56] Novák P., Michalcová A., Marek I., Mudrová M., Saksl K., Bednarčík J., Zikmund P., Vojtěch D. (2013). On the formation of intermetallics in Fe Al system an in situ XRD study. Intermetallics.

[57] Wiessner M., Angerer P., Van der Zwaag S., Gamsjäger E. (2021). Transient Phase Fraction and Dislocation Density Estimation from In-Situ X-Ray Diffraction Data with a Low Signal-to-Noise Ratio Using a Bayesian Approach to the Rietveld Analysis. Mater. Charact..

[58] Karagöz S., Fischmeister H.F. (1998). Cutting Performance and Microstructure of High Speed Steels: Contributions of Matrix Strengthening and Undissolved Carbides. Met. Mater. Trans. A.

[59] Li K., Yu B., Misra R.D.K., Han G., Liu S., Shang C.J. (2018). Strengthening of cobalt-free 19Ni3Mo1.5Ti maraging steel through high-density and low lattice misfit nanoscale precipitates. Mater. Sci. Eng. A.

[60] Fathy A., Mattar T., EI-Faramawy H., Bleck W. (2002). Mechanical properties of new low-nickel cobalt-free maraging steels. Steel Res..

[61] Cheng L., Böttger A., De Keijser T.H., Mittemeijer E.J. (1990). Lattice parameters of iron-carbon and iron-nitrogen martensites and austenites. Scr. Metall. Mater..

[62] Krisement O. (1957). Kalorimetrische Untersuchungen zur Kinetik des Martensitanlassens. Archiv für Eisenhüttenwesen.

[63] Dobrzanski L.A., Zarychta A., Ligarski M. (1997). High-Speed Steels with Addition of Niobium or Titanium. J. Mater. Process. Technol..

[64] Mirzaee M., Momeni A., Keshmiri H., Razavinejad R. (2014). Effect of Titanium and Niobium on Modifying the Microstructure of Cast K100 Tool Steel. Met. Mater. Trans. B.

[65] Pavlickova M., Vojtech D., Stolar P., Jurci P. (2002). Properties of rapidly solidified niobium-alloyed tool steel. Kovove Materialy.

[66] Pavlíčková M., Vojtěch D., Novák P., Gemperlová J., Gemperle A., Zárubová N., Jurči P., Lejček P. (2004). Influence of Thermal Treatment on Microstructure and Hardness of Niobium Alloyed PM/Tool Steel. Instrum. Sci. Technol..

[67] Novák P., Vojtěch D., Šerák J., Knotek V., Bartová B. (2006). Duplex surface treatment of the Nb-alloyed PM tool steel. Surf. Coatings Technol..

[68] Shim K.H., Lee S.K., Kang B.S., Hwang S.M. (2004). Investigation of blanking of thin sheet metal using ductile fracture criterion and its experimental verification. J. Mater. Process. Technol..

[69] Monteil G., Greban F., Roizard X. (2008). In situ punch wear measurement in a blanking tool by means of thin layer activation. Wear.

[70] Mayrhofer P.H., Mitterer C., Hultman L., Clemens H. (2006). Microstructural design of hard coatings. Prog. Mater. Sci..

[71] Hovsepian P.E., Lewis D.B., Münz W.-D. (2000). Recent progress in large scale manufacturing of multilayer/superlattice hard coatings. Surf. Coat. Technol..

[72] Tkadletz M., Schalk N., Daniel R., Keckes J., Czettl C., Mitterer C. (2016). Advanced characterization methods for wear resistant hard coatings: A review on recent progress. Surf. Coat. Technol..

[73] Nguyen T.D., Kim S.K., Lee D.B. (2009). High-temperature oxidation of nano-multilayered TiAlCrSiN thin films in air. Surf. Coatings Technol..

[74] Kalss W., Reiter A., Derflinger V., Gey C., Endrino J.L. (2006). Modern coatings in high performance cutting applications. Int. J. Refract. Met. Hard Mater..

[75] Endrino J.L., Derflinger V. (2005). The influence of alloying elements on the phase stability and mechanical properties of AlCrN coatings. Surf. Coatings Technol..

[76] Kim D.G., Seong T.Y., Baik Y.J. (2002). Effects of annealing on the microstructures and mechanical properties of TiN/AlN nano-multilayer films prepared by ion-beam assisted deposition. Surf. Coat. Technol..

[77] Shinn M., Hultman L., Barnett S.A. (1992). Growth, structure and microhardness of epitaxial TiN/ NbN superlattices. J. Mater. Res..

[78] Ali F., Park B.S., Kwak J.S. (2013). Effect of number of bi-layers on properties of TiN/TiAlN multilayer coatings. J. Ceram. Process Res..

[79] Yang Q., He C., Zhao L.R., Immarigeon J.P. (2002). Preferred orientation and hardness enhancement of TiN/CrN superlattice coatings deposited by reactive magnetron sputtering. Scr. Mater..

[80] Lin J., Moore J.J., Mishra B., Pinkas M., Zhang X., Sproul W.D. (2009). CrN/AlN superlattice coatings synthesized by pulsed closed field unbalanced magnetron sputtering with different CrN layer thicknesses. Thin Solid Films.

[81] Reiter A.E., Derflinger T.V.H., Hanselmann B., Bachmann T., Sartory B. (2005). Investigation of the properties of Al1-xCrxN coatings prepared by cathodic arc evaporation. Surf. Coat. Technol..

[82] Jakubéczyová D., Hvizdoš P., Selecká M. (2012). Investigation of thin layers deposited by two PVD techniques on high speed steel produced by powder metallurgy. Appl. Surf. Sci..

[83] Cabibbo M., Ricci P., Cecchini R., Rymuza Z., Sullivan J., Dub S., Cohen S. (2012). An international round-robin calibration protocol for nanoindentation measurements. Micron.

[84] Cabibbo M., Clemente N., El Mehtedi M., Hamouda A.H., Musharavati F., Santecchia E., Spigarelli S. (2015). Constitutive analysis for the quantification of hardness decay in a superlattice CrN/NbN hard-coating. Surf. Coat. Technol..

[85] Santecchia E., Hamouda A.M.S., Musharavati F., Zalnezhad E., Cabibbo M., Spigarelli S. (2015). Wear resistance investigation of titanium nitride-based coatings. Ceram. Int. Part A.

[86] Fabrizi A., Cecchini R., Kiryukhantsev-Korneev P.V., Sheveyko A.N., Spigarelli S., Cabibbo M. (2017). Comparative investigation of oxidation resistance and thermal stability of nanostructured Ti-B-N and Ti-Si-B-N coatings. Prot. Met. Phys. Chem. Surf..

[87] Santecchia E., Cabibbo M., Hamouda A.M.S., Musharavati F., Popelka A., Spigarelli S. (2019). Investigation of the Temperature-Related Wear Performance of Hard Nanostructured Coatings Deposited on a S600 High Speed Steel. Metals.

[88] Kawate M., Hashimoto A.K., Suzuki T. (2003). Oxidation resistance of Cr1-xAlxN and Ti1-xAlxN films. Surf. Coat. Technol..

[89] Choi P.-P., Povstugar I., Ahn J.-P., Kostka A., Raabe D. (2011). Thermal stability of TiAlN/CrN multilayer coatings studied by atom probe tomography. Ultramicroscopy.

[90] Barshilia H., Prakash M.S., Jain A., Rajam K.S. (2005). Structure, hardness and thermal stability of TiAlN and nanolayered TiAlN/CrN multilayer films. Vacuum.

[91] Forsén R., Johansson M.P., Odén M., Ghafoor N. (2013). Effects of Ti alloying of AlCrN coatings on thermal stability and oxidation resistance. Thin Solid Films.

[92] Beake B.D., Fox-Rabinovich G.S. (2014). Progress in high temperature nanomechanical testing of coatings for optimising their performance in high speed machining. Surf. Coat. Technol..

[93] (2014). 48th Census of World Casting Production. Modern. Cast..

[94] Sidjanin L., Smallman E.R., Young J.M. (1994). Electron Microstructure and Mechanical Properties of Silicon and Aluminium Ductile Irons. Acta Met. Mater..

[95] Sidjanin L., Rajnovic D., Eric O., Smallman R.E. (2010). Austempering study of unalloyed and alloyed ductile irons. Mater. Sci. Technol..

[96] Eric O., Sidjanin L., Rajnovic D., Balos S. (2014). The Austempering Kinetics of Cu-Ni Alloyed Austempered Ductile Iron. Met. Mater. Int..

[97] Rajnovic D., Eric O., Sidjanin L. (2012). The standard processing window of alloyed ADI materials. Kovove Mater..

[98] Rajnovic D., Eric O., Sidjanin L. (2008). Transition temperature and fracture mode of as-cast and austempered ductile iron. J. Microsc..

[99] Martinez R.A. (2010). Fracture surfaces and the associated failure mechanisms in ductile iron with different matrices and load bearing. Eng. Fract. Mech..

[100] Eric O., Rajnović D., Zec S., Sidjanin L., Jovanović T. (2006). Microstructure and fracture of alloyed austempered ductile iron. Mater. Charact..

[101] Harding R.A. (2007). The production, properties and automotive applications for austempered ductile iron. Kovove Mater..

[102] Goergen F., Mevissen D., Masaggia S., Veneri E., Brimmers J., Brecher C. (2020). Contact Fatigue Strength of Austempered Ductile Iron (ADI) in Gear Applications. Metals.

[103] Balos S., Rajnovic D., Dramicanin M., Labus D., Cekic O.E., Grbovic-Novakovic J., Sidjanin L. (2016). Abrasive wear behaviour of ADI material with various retained austenite content. Int. J. Cast Metals Res..

[104] Dojcinovica M., Cekic O.E., Rajnovic D., Sidjanin L., Balos S. (2013). Effect of austempering temperature on cavitation behaviour of unalloyed ADI material. Mater. Charact..

[105] Rajnovic D., Balos S., Sidjanin L., Cekic O.E., Grbovic Novakovic J. (2015). Tensile properties of ADI material in water and gaseous environments. Mater. Charact..

[106] Janjatovic P., Cekic O.E., Sidjanin L., Balos S., Dramicanin M., Grbovic Novakovic J., Rajnovic D. (2021). The Effect of Water Concentration in Ethyl Alcohol on the Environmentally Assisted Embrittlement of Austempered Ductile Irons. Metals.

[107] Balos S., Radisavljevic I., Rajnovic D., Dramicanin M., Tabakovic S., Cekic O.E., Sidjanin L. (2015). Geometry, mechanical and ballistic properties of ADI material perforated plates. Mater. Des..

[108] Balos S., Radisavljevic I., Rajnovic D., Janjatovic P., Dramicanin M., Eric-Cekic O., Sidjanin L. (2019). Ballistic Behaviour of Austempered Compacted Graphite Iron Perforated Plates. Def. Sci. J..

[109] Novák P., Vanka T., Nová K., Stoulil J., Průša F., Kopeček J., Haušild P., Laufek F. (2019). Structure and Properties of Fe–Al–Si Alloy Prepared by Mechanical Alloying. Materials.

[110] Ringwood A.E. (1990). Diamond Compacts and Process for Making Same. U.S. Patent.

[111] Jaworska L. (2011). Diamond-Ceramic Bonding Phase Composites for Application in Cutting Tools. Ceram. Mater..

[112] Cygan S., Jaworska L., Putyra P., Ratuszek W., Cyboroń J., Klimczyk P. (2017). Thermal Stability and Coefficient of Friction of the Diamond Composites with the Titanium Compound Bonding Phase. J. Mater. Eng. Perform..

[113] Tönshoff H.K., Hillmann­Apmann H., Asche j. (2002). Diamond tools in stone and civil engineering industry: Cutting principles, wear and applications. Diam. Relat. Mater..

[114] Dormishi A., Ataei M., Mikaeil R., Khalokakaei R., Haghshenas S.S. (2019). Evaluation of gang saws’ performance in the carbonate rock cutting process using feasibility of intelligent approaches. Eng. Sci. Technol. Int. J..

[115] Ersoy A., Atici U. (2004). Performance characteristics of circular diamond saws in cutting different types of rocks. Diam. Relat. Mater..

[116] Konstanty J. (2006). Production parameters and materials selection of powder metallurgy diamond tools. Powder Metall..

[117] Büttner A. (1974). Diamond tools and stone. Ind. Diam. Rev..

[118] Chalus P.A.D. (1994). Metal powders for optimum grain retention. Ind. Diam. Rev..

[119] Bullen G.J. (1982). Choosing the best grit for the job. Ind. Diam. Rev..

[120] Konstanty J. (1991). The materials science of stone sawing. Ind. Diam. Rev..

[121] Wright D.N., Tagg W.R.J. (1998). The development of a rock classification system for use with diamond tools. Ind. Diam. Rev..

[122] Molinari A., Marchetti F., Gialanella S., Scardi P., Tiziani A. (1990). Study of the Diamond-Matrix Interface in Hot-pressed Cobalt-based Tools. Mater. Sci. Eng. A.

[123] Hsieh Y.Z., Lin S.T. (2001). Diamond tool bits with iron alloys as the binding matrixes. Mater. Chem. Phys..

[124] Spriano S., Chen Q., Settineri L., Bugliosi S. (2005). Low content and free Cobalt matrixes for diamond tools. Wear.

[125] Del Villar M., Muro P., Sanchez J.M., Iturriza I., Castro F. (2001). Consolidation of diamond tools using Cu-Co-Fe based alloys as metallic binders. Powder Metall..

[126] Lison D., Buchet J.P., Swennen B., Molders J., Lauwerys R. (1994). Biological monitoring of workers exposed to cobalt metal, salt, oxides, and hard metal dust. Occup. Environ. Med..

[127] Goerting K., Brewin P. European New Chemicals Policy Response of The Hard Materials Industry. Proceedings of the European Conference on Hard Materials and Diamond Tooling—Euro PM 2002, EPMA.

[128] Weber G., Weiss C. (2005). DIAMIX—A family of bonds based on DIABASE-V21. Ind. Diam. Rev..

[129] Bonneau M. (1999). NEXT and NEXT Pre-mixed Powders. Diam. Appl. Technol..

[130] Clark I.E. (2002). Cobalite HDR-a new prealloyed matrix powder for diamond construction tools. Ind. Diam. Rev..

[131] Eurotungstene (2005). Keen®—A new concept in prealloyed powders. Ind. Diam. Rev..

[132] Kamphuis B., Serneels A. (2004). Cobalt and nickel free bond powder for diamond tools: Cobalite^®^ CNF. Ind. Diam. Rev..

[133] De Oliveira H.C.P., Cabral S.C., Guimaries R.S., Bobrovnitchii G.S., Filgueira M. (2009). Processing and characterization of a cobalt based alloy for use in diamond cutting tools. Materialwissenschaft.

[134] Palumbo M., Curiotto S., Battezzati L. (2006). Thermodynamic analysis of the stable and metastable Co-Cu and Co-Cu-Fe phase diagrams. Calphad.

[135] Huang X., Mashimo T. (1999). Metastable BCC and FCC alloy bulk bodies in Fe-Cu system prepared by mechanical alloying and shock compression. J. Alloys Compd..

[136] Gaffet E., Harmelin M., Faudot F. (1993). Far-from-equilibrium phase transition induced by mechanical alloying in the Cu-Fe system. J. Alloys Compd..

[137] Menapace C., Bocchi E., Costa P., Molinari A. Microstructural and mechanical characterization of iron and copper based powders for diamond tools. Proceedings of the 2004 Powder Metallurgy World Congress, European Powder Metallurgy Association.

[138] Menapace C., Costa P., Molinari A. Wear and Cutting Properties of New Diamond Inserts Based on Iron and Copper Powders. Proceedings of the European Powder Metallurgy Congress and Exhibition.

[139] De Oliveira L.J., Bobrovnitchii G.S., Filgueira M. (2007). Processing and characterization of impregnated diamond cutting tools using a ferrous metal matrix. Int. J. Refract. Hard Met..

[140] Meszaros M., Vadasdi K. (1994). Process and equipment for electrochemical etching of Diamond-containing Co-WC tools and recovery of diamond from used steel tools. Int. J. Refract. Metals Hard Mater..

[141] Baroura L., Boukhobza A., Derardja A., Fedaoui K. (2018). Study of Microstructure and Mechanical Properties of Sintered Fe-Cu Alloys. Int. J. Eng. Res. Afr..

[142] Sung C.M., Tai M.F. (1997). Reactivities of transition metals with carbon: Implications to the mechanism of diamond synthesis under high pressure. Int. J. Refract. Hard Met. Hard Mater..

[143] Tillmann W., Ferreira M., Steffen A., Rüster K., Möller J., Bieder S., Paulus M., Tolan M. (2013). Carbon reactivity of binder metals in diamond-metal composites—Characterization by scanning electron microscopy and X-ray diffraction. Diam. Relat. Mater..

[144] Jaworska L., Szutkowska M., Klimczyk P., Sitarz M., Bucko M., Rutkowski P., Figiel P., Lojewska J. (2014). Oxidation, graphitization, and thermal resistance of PCD materials with the various bonding phases of up to 800 °C. Int. J. Refract. Met. Hard Mater..

[145] De Oliveira L.J., Cabral S.C., Filgueira M. (2015). Study of the TiC Coating on Powder Metallurgy Diamonds Tool’s Performance. Mater. Res..

[146] Borowiecka-Jamrozek J., Lachowski J. Modelling of retention of a diamond particle in matrices based on Fe and Cu. Proceedings of the XXI International Polish-Slovak Conference “Machine Modeling and Simulations”.

[147] Borowiecka-Jamrozek J., Konstanty J., Lachowski J. (2018). The application of a ball-milled Fe-Cu-Ni powder mixture to fabricate sintered diamond tools. Arch. Foundry Eng..

[148] Konstanty J., Romański A., Baczek E., Tyrala D. (2015). New Wear Resistant Iron-Base Matrix Materials for The Fabrication of Sintered Diamond Tools. Arch. Met. Mater..

[149] Konstanty J., Romanski A. (2012). New nanocrystalline Matrix Materials for Sintered Diamond Tools. Mater. Sci. Appl..

[150] Borowiecka-Jamrozek J. (2017). Sintered Fe-Cu-Re alloys produced from commercially available powders. Arch. Met. Mater..

[151] Mechnik V.A., Bondarenko N.A., Kolodnitskyi V.M., Zakiev V.I., Zakiev I.M., Ignatovich S.R., Yutskevych S.S. (2020). Mechanical and Tribological Properties of Fe-Cu-Ni-Sn Materials with Different Amounts of CrB2 Used as Matrices for Diamond-Containing Composites. J. Superhard Mater..

[152] Tyrala D., Romanski A., Konstanty J. (2020). The Effects of Powder Composition on Microstructure and Properties of Hot-Pressed Matrix Materials for Sintered Diamond Tools. J. Mater. Eng. Perform..

[153] Loginov P.A., Sidorenko D.A., Bychkova M.Y., Zaitsev A.A., Levashov E.A. (2020). Performance of diamond drill bits with hybrid nanoreinforced Fe-Ni-Mo binder. Int. J. Adv. Manuf. Technol..

[154] Kratochvíl P. (2008). The history of the search and use of heat resistant Pyroferal^©^ alloys based on FeAl. Intermetallics.

[155] Vodičková V., Švec M., Hanus P., Novák P., Záděra A., Keller V., Prokopčáková P.P. (2020). The Effect of Simultaneous Si and Ti/Mo Alloying on High-Temperature Strength of Fe3Al-Based Iron Aluminides. Molecules.

[156] Novák P., Nová K. (2019). Oxidation Behavior of Fe-Al, Fe-Si and Fe-Al-Si Intermetallics. Materials.

[157] Novák P., Zelinková M., Šerák J., Michalcová A., Novák M., Vojtěch D. (2011). Oxidation resistance of SHS Fe-Al-Si alloys at 800 °C in air. Intermetallics.

[158] Novák P., Jaworska L., Cabibbo M. (2018). Intermetallics as innovative CRM-free materials. IOP Conference Series: Mater. Sci. Eng..

[159] Šerák J., Vojtěch D., Novák P., Šefl V., Janoušek T. (2008). Možnosti snížení obsahu železa ve slitinách AlSiCuMgFe. Slévárenství.

